# Prevalence and associated factors of metabolic body size phenotype in children and adolescents: A national cross-sectional analysis in China

**DOI:** 10.3389/fendo.2022.952825

**Published:** 2022-08-25

**Authors:** Jieyu Liu, Tao Ma, Manman Chen, Ying Ma, Yanhui Li, Di Gao, Qi Ma, Xinxin Wang, Li Chen, Yi Zhang, Yanhui Dong, Yi Song, Jun Ma

**Affiliations:** ^1^ Institute of Child and Adolescent Health, School of Public Health, Peking University, Beijing, China; ^2^ National Health Commission Key Laboratory of Reproductive Health, Beijing, China; ^3^ School of Public Health and Management, Ningxia Medical University, Yinchuan, China

**Keywords:** metabolic health, obesity, children and adolescents, prevalence, associated factors

## Abstract

**Background:**

Metabolically healthy obesity (MHO) is a group of subjects with overweight/obesity who present a metabolically healthy profile; however, associated factors are complex and are far from completely understood. The aim of the current study was to estimate the prevalence of different metabolic body size phenotypes and investigate the associated factors in Chinese children and adolescents.

**Methods:**

A cross-sectional survey was conducted of 12,346 children and adolescents aged 7–18 years from seven provinces in China in 2013. Anthropometric, blood pressure, and biochemical measurements were obtained. A multi-component questionnaire covering demographic, neonatal, and lifestyle characteristics was administered. The classification of metabolic body size phenotype based on three definitions was compared. With metabolically healthy with normal weight (MHNW) as a reference group, logistic regression analyses were used to estimate the potential effects of associated risk factors, with adjustment for age, sex, single-child status, and residence area.

**Results:**

The prevalence of MHNW, MHO, metabolically unhealthy with normal weight (MUNW), and metabolically unhealthy overweight/obesity (MUO) phenotype was 68.6%, 2.0%, 26.4%, and 3.0%, respectively. There were 39.3% MHO and 60.7% MUO among obese participants and 72.2% MHNW and 27.8% MUNW among those with normal weight. Compared to cardiometabolic risk factor (CMRF) criteria and metabolic syndrome (MetS) component definition, the application of the 2018 consensus-based definition may identify more children with abnormal cardiovascular risks, independent of weight status. Compared to younger children, older-aged adolescents were positively associated with higher risks of MUNW (odds ratio (OR) = 1.38, 95% CI = 1.27–1.50) and MUO (OR = 1.29, 95% CI = 1.04–1.60), while factors positively associated with MHO were younger age, single-child status, urban residence, high birth weight, prolonged breastfeeding duration, parental overweight/obesity status, long screen time, and less physical activity.

**Conclusion:**

There were still a high proportion of children and adolescents at high cardiometabolic risk in China. Our findings reinforce the need for cardiometabolic risk prevention in children and adolescents irrespective of their weight statuses, such as parental educational programs and healthy lifestyle interventions.

## Introduction

Childhood obesity is a complex, chronic disease influenced by biological, behavioral, and environmental factors. The crude prevalence in children and adolescents aged 5 to 19 years more than doubled (from 2.9% to 6.8%) worldwide since 2016 (1). It is well acknowledged that childhood obesity is associated with higher chances of metabolic disorders including diabetes, hypertension, dyslipidemia, and cardiovascular disease (2); however, not all the metabolic body size phenotypes exhibit complications to the same severity and extent. As a phenotype of obesity, metabolically healthy obesity (MHO) is defined as a condition in which, despite the significant excess weight, traditional risk factors such as insulin resistance (IR), dyslipidemia, and hypertension are not present (3, 4), contrary to what occurs in the metabolically unhealthy obesity (MUO) condition. MUO is widely studied in pediatric patients because of its devastating consequences in adulthood (5), such as worse insulin sensitivity and higher fasting plasma glucose and triglyceride concentrations (6). Norbert Stefan and colleagues confirmed that metabolically healthy but obese people had a better ability to trap free fatty acids in adipose tissue. Additionally, these people had lower intima-media thickness in the carotid artery and a favorable cardiovascular profile (7). However, metabolically benign obesity should not be considered a safe condition. In addition to metabolic and cardiovascular diseases, obesity is also associated with osteoarthritis, back pain, asthma, depression, cognitive impairment, and some types of cancer (8). In addition, some participants with normal weight have a variety of metabolic disorders; these individuals are defined as the metabolically unhealthy normal weight (MUNW) phenotype (9). However, the normal weight will always cover up their need for timely intervention; early identification of the MUNW population therefore becomes particularly important.

Once the MHO and MUNW phenotypes were proposed, they gained much attention from scholars. There is still no universally accepted definition of childhood metabolic body size phenotype (6). MHO and MUO are differentiated by the presence of cardiometabolic risk factors (CMRFs) and IR, and the prevalence of MHO (CMRF) and MHO (IR) in obese Korean youth was 36.8% and 68.8%, respectively (10). Based on metabolic syndrome (MetS) components and IR criteria, the prevalence of MHO phenotype varied from 49.4% to 55.9% (11). The findings showed a higher prevalence than those found in European (35.4%), North American (37.6%), and Asian (35.4%) adolescent-based studies that used the same criteria for MHO phenotype (10, 12, 13). In China, the prevalence of childhood metabolic body size phenotype varied due to regional differences. The overall prevalence rates of metabolically unhealthy with normal weight (MUNW) and MHO were 10.6% and 15.3% in urban areas in seven cities (Beijing, Changchun, Jinan, Yinchuan, Shanghai, Chongqing, and Chengdu) (14). However, that prevalence in Ningxia was higher for MUNW (38.7%) and lower for MHO (7.1%). However, they only concern adolescent ages (10–18 years) (15). In view of the current unsatisfactory situation that the peak of obesity rates is trending increasingly toward younger ages and obesity brings a heavy economic burden, especially in developing countries like China, there is still a lack of effective assessment of nationally representative data to estimate the burden of childhood metabolic body size types.

Notably, associated factors of different metabolic body size phenotypes are far from completely understood. Older age or male sex seemed to be not associated with MUO among Mexican children (16), but the opposite was true for Turkish children (17). For lifestyle behaviors, longer vigorous physical activity and consumption of soft drinks were associated with childhood MHO phenotype (10, 18), while a sedentary lifestyle was correlated with MUO (17). However, there were no significant differences in physical activity between MHO and MUO in U.S. adolescents (19). Additionally, a few studies showed that the factors (e.g., age, sex, and ethnicity) associated with MHO and MUNW were not similar (20, 21). Differences might be explained by ethnicity, heterogeneity of the study population, study design, or other residual confounders. There is still inadequate evidence identifying determinants and modifiable risk factors for the better prevention of conversions from MHO to MUO and cardiometabolic disease manifestations, based on large-scale national-level data of children and adolescents.

In 2018, a scoping review was carried out in order to reach a consensus-based definition of pediatric MHO through experts’ consultation and the application of a Delphi process (22); the experts agreed on applying the World Health Organization body mass index (BMI) criteria to assess weight status and including high-density lipoprotein cholesterol (HDL-C), triglycerides (TG), glycemia, and blood pressure (systolic blood pressure (SBP) and diastolic blood pressure (DBP)) to define MHO status. However, there was no finding evaluating the consensus-based criteria in the Chinese population. For this reason, based on a cross-sectional survey conducted in seven provinces in China, we aimed to 1) determine the prevalence of metabolic body size phenotype among Chinese children and adolescents with greater age diversity (between 7 and 18 years old), according to the consensus-based pediatric MHO definition (23), and compare it with other widely used definitions, and 2) further assess the potential effects of modifiable factors such as demographic, socioeconomic, dietary, and other lifestyle behaviors.

## Methods

### Study population

**Figure 1:**
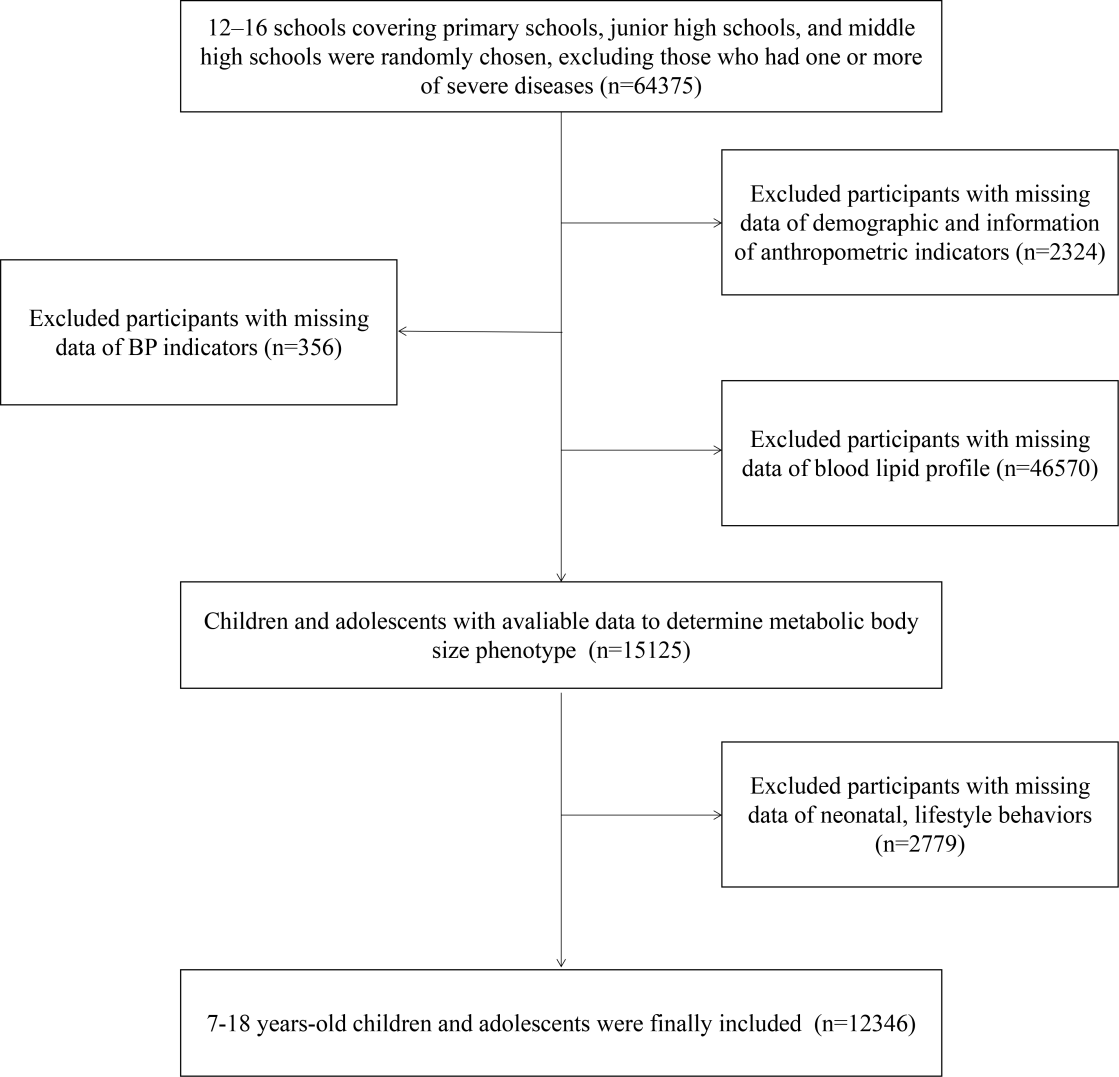
Flow diagram of study population selection.

The Health Lifestyles Intervention in Chinese Children and Adolescents (HLI-CCA) was a multicenter cluster non-randomized controlled school-based intervention aiming to prevent childhood obesity, which was conducted from September 2013 to February 2014. Data in this study came from the baseline of the trial, including children and adolescents from seven provinces or cities of China (Hunan, Ningxia, Tianjin, Chongqing, Liaoning, Shanghai, and Guangzhou; registration number: NCT02343588). The full trial protocol has been presented elsewhere (24, 25). Briefly, based on a multi-stage cluster random sampling method, several regions were randomly chosen from each province or city, and approximately 12–16 schools (including primary schools, junior high schools, and middle high schools) were chosen randomly from each region. In the selected schools, two classes from each grade were randomly selected, and all students and parents were invited to participate. According to the inclusion and exclusion criteria, students who had one or more of the following conditions were excluded: 1) serious organ disease (e.g., heart, lung, and liver), 2) abnormal physical development (e.g., pygmyism or gigantism), 3) physical impairment or deformity (e.g., severe scoliosis, pectus carinatum, and limp), 4) and acute disease symptoms (e.g., diarrhea and high fever) during the past month and not yet recovered. Furthermore, participants with missing data on demographic factors and measurements of anthropometric, blood pressure (BP), and lipid profiles were excluded. Finally, a total of 12,346 participants aged 7–18 years whose physical examination and blood samples were available were included. The flow diagram of study population selection is presented in **Figure 1**. The study has been approved by the ethical committee of Peking University (number: IRB0000105213034). Written informed consent was obtained from both students and their parents or legal guardian.

### Anthropometric parameters, blood pressure, and blood sample collection

Anthropometric measurements were conducted by trained project members following the standardized procedure. Children were required to stand straight in light clothing and without shoes. Height was measured with an accuracy of 0.1 cm using a portable stadiometer (model TZG, Jiangyin Hongya Science and Education Equipment Co., Ltd., Jiangyin, China); weight was measured to the nearest 0.1 kg by a lever-type weight scale (model RGT-140, China). Waist circumference (WC) was measured with an accuracy of 0.1 cm using a non-elastic tape at the end of a natural breath at the midpoint between the top of the iliac crest and the lower margin of the last palpable rib. Every indicator was measured twice, and the average level of the two measurements was used for final analyses. BMI was calculated as body weight (kg) divided by height (m) squared.

BP was measured using an auscultation mercury sphygmomanometer (model XJ1ID, China) with an appropriate cuff for children. Three cuff sizes (7-, 9-, and 12-cm width) were selected according to the mid-upper arm circumference of the children, and the cuff bladder width should cover 50%–75% of the mid-arm circumference. The cuff was placed ~2 cm above the crease of the elbow. The child was asked to seat comfortably for at least 10 min prior to the first reading. Blood pressure was measured twice, with a 1-min break between each measurement. SBP was determined by the onset of the first Korotkoff sound (K1), and DBP was determined by the fifth Korotkoff sound (K5). The stadiometers, lever-type weight scales, non-elastic tape, and auscultation mercury sphygmomanometer were all calibrated, and the measuring instruments were similar at all investigated schools.

Venous blood samples were obtained in the morning after overnight (at least 8 h) fasting. Children were required to rest for at least 10 min prior to blood sample collection. Blood specimens were transported in a chilled insulated container immediately, centrifuged at 3,000 rpm for 10 min, and then frozen at −80°C. All plasma samples were transported in dry ice to the laboratory in Beijing, where the samples were stored at −80°C before laboratory detection. All the biochemical analyses were conducted at a biomedical analyses company accredited by Peking University (24). Lipid profiles were measured with an autoanalyzer (TBA-120FR, Toshiba, Tokyo, Japan), with TG assayed by enzymatic method, while HDL-C was measured by clearance method.

### Questionnaire

The children’s questionnaire was used to collect basic information and lifestyle behaviors. Furthermore, the parental self-administrated questionnaire included information about demographic, neonatal, parental, or family characteristics. To obtain more accurate information, both parental and children’s questionnaires of children grades 1–3 were reported to parents. Children above the fourth grade would fill in children’s questionnaires instructed by a trained teacher.

For demographic factors, single-child status was classified into “yes” or “no”. Residence area was divided into “rural area” and “urban area”. As for neonatal features, low birth weight was described as an infant with a weight of less than 2,500 g at delivery, and high birth weight was defined as an infant with a weight heavier than 4,000 g at delivery (26). Parents were also asked to provide information on feeding type (breastfeeding or not), as well as the duration of breastfeeding (in months), which were divided into non-breastfeeding, 0–6, 6–12, and >12 months (27). For parental or family characteristics, parents were asked to report their height (cm) and weight (kg), while BMI was calculated as the weight (kg) divided by the square of the height (m^2^). According to the criteria established by the Working Group on Obesity in China (WGOC) for Chinese adults (28), BMI cutoffs of 24 and 28 kg/m^2^ were applied to categorize normal, overweight, and obesity. Parental educational attainment was grouped into “primary school or below”, “secondary or equivalent”, and “junior college or above”, and monthly household income was defined as the sum of monthly income (in CNY) of all household members and then divided into <5,000, 5,000–12,000, or ≥12,000 CNY.

For dietary behaviors, the frequency (days) and amount (serving per day) of the consumption of fruits, vegetables, and sugar-sweetened beverages (SSBs) over the past 7 days were investigated, as previously published (24, 29, 30). Participants were asked, “How many days have you eaten fruit/vegetables or drunk SSB over the past 7 days? How many servings in one day?”. One serving of fruit/vegetable was defined as the size of an ordinary adult’s closed fist and roughly equaled a medium-sized apple (approximately 200 g) (31), which has been described elsewhere (30). SSB included Coca-Cola, Sprite, orange juice, Nutrition Express, and Red Bull (32). One serving of SSB was determined as a canned beverage (approximately 250 ml). The dietary intake was calculated as average daily intake = (days of consumption × servings in those days)/7.

Sedentary behavior was categorized by the total time per day when participants spent sitting, reading, or doing homework, except for lying on the bed. Screen time was defined according to the total time spent on watching electronic devices and playing electronic games and classified into “<1 h/day”, “1–3 h/day”, and “≥3 h/day”. In addition, they were asked to report their average daily sleep duration for the past 7 days, and they were divided into “<7 h”, “7–9 h”, and “≥9 h”. Information about the child’s physical activity was collected using the International Physical Activity Questionnaire-Short Form (IPAQ-SF) (33). All recruited participants reported the frequency (days) and duration (hours and minutes) of moderate-to-vigorous-intensity physical activities (MVPAs) over the past 7 days, and the average time for MVPA per day was calculated as average daily time = (days for MVPA × duration in those days)/7.

### Outcome definitions

According to the Working Group on Obesity in China (34), childhood obesity was defined using the age- and sex-specific BMI standards (BMI ≥the cutoffs of 95th percentile).

Three criteria were used to define metabolic body size status in the current study—CMRF criteria (35), MetS component criteria (36), and the 2018 consensus-based criteria (22, 23)—which have been widely used in children and adolescents worldwide (determined by the cutoff points for the current children and adolescents). The detailed cutoff values of each component in different criteria are presented in **Table 1**. According to the CMRF criteria defined by the International Diabetes Federation, obese subjects without any CMRF were defined as having MHO, and those with one or more CMRF were defined as having MUO by risk factors (37). In the MetS component definition, MHO was defined as central obesity with <2 MS components and MUO as central obesity with ≥2 MS components. Based on the 2018 consensus-based criteria, the definition lacked consensus on what measure of glycemia should be used. Since most of the studies reviewed by the expert consensus used fasting glucose <100 mg/dl, we used the same value in our study. MHO subjects were classified as obese without the above risk factors, and MUO subjects were classified as obese with at least one of the risk factors.

**Table 1:** Detailed cutoff values of each component in three MHO criteria.

Cardiometabolic risk factors	CMRF criteria	MetS component criteria	2018 consensus-based criteria
BMI	≥95th percentile	\	≥95th percentile
WC	\	≥90th percentile	\
SBP or DBP	≥130/85 mmHg	>90th percentile	>90th percentile
Fasting glycemia (mmol/L)	≥5.6	≥5.6	≥5.6
TG (mmol/L)	≥1.7	≥1.24	≥1.7
HDL cholesterol (mmol/L)	≤1.03 in boys and girls aged under 15 years; ≤1.29 in girls aged 16–18 years	≤1.03	≤1.03

CMRF, cardiometabolic risk factor; MetS, metabolic syndrome; BMI, body mass index; WC, waist circumference; SBP, systolic blood pressure; DBP, diastolic blood pressure; HDL-C, high-density lipoprotein cholesterol; TG, triglycerides; MHO, metabolically healthy obesity.

### Statistical analysis

Continuous variables were expressed as mean ± standard deviation (SD), and categorical variables were expressed as numbers and percentages. Differences in demographic, neonatal, parental or family, and lifestyle characteristics by metabolic body size phenotype were examined using one-way analysis of variance (ANOVA) for continuous variables and Pearson’s chi-squared test for categorical variables. The region-weighted rate was also calculated by using the national population proportion, based on the 2020 national census data (**Supplementary Table 1**). In addition, the distribution of cardiometabolic risk factors among metabolic body size groups was investigated. The percentages of four phenotypes were presented according to age, sex, single-child status, and residence area, presented by percent stacked bar charts and column charts. Logistic regression was used to calculate the odds ratio (OR) and 95% confidence interval (95% CI), to analyze the potential factors associated with MHO, MUNW, and MUO, with the MHNW individuals as the reference group. The model was adjusted for age, sex, single-child status, and residence area. All statistical analyses were performed using Statistical Analysis System (SAS) software (version 9.4, SAS Institute, Cary, NC, USA), and a two-sided *p* < 0.05 was considered statistically significant.

## Results

### Baseline characteristics of study population

**Figure 2:**
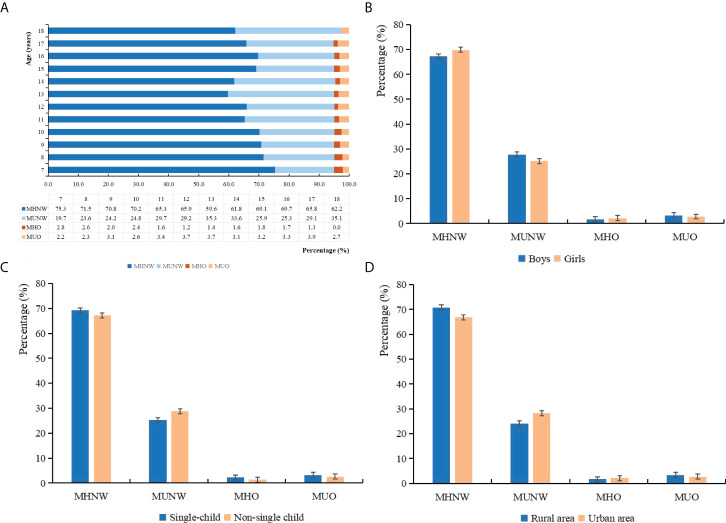
Percentage of different metabolic obesity phenotypes among each demographic group: **(A)** age, **(B)** sex, **(C)** single-child status, and **(D)** residence area.

Based on the 2018 consensus-based criteria, the prevalence of MHNW, MUNW, MHO, and MUO phenotypes was 68.6%, 26.4%, 2.0%, and 3.0%, respectively (**Table 2**). Notably, 39.3% and 60.7% of obese individuals were MHO and MUO, respectively, among all obese children and adolescents. There were more boys than girls in the group of MUNW (boys *vs.* girls, 52.60% *vs.* 47.40%) and MUO (boys *vs.* girls, 54.01% *vs.* 45.99%). Metabolically unhealthy groups seemed to have lower proportions of well-educated parents, and the family of MUO individuals tended to have lower monthly household income (all *p* < 0.001). Also, they were more likely to consume more SSB and have shorter sleep duration (*p* < 0.001). Notably, with increasing age, the prevalence of MHNW was decreasing, and the prevalence of MUNW was increasing. Boys, single children, and individuals who came from rural areas tended to have more MUO phenotypes (**Figure 2**).

**Table 2:** Baseline characteristic of included population among different metabolic obesity phenotypes, based on 2018 consensus-based criteria.

Characteristics	MHNW (n = 8,466)	MUNW (n = 3,264)	MHO (n = 242)	MUO (n = 374)	*p*-Value
**Demographic factors**					
Age, year	11.20 ± 3.14	11.62 ± 2.99	10.65 ± 3.12	11.73 ± 3.02	<0.0001
Boys, n (%)	4,165 (49.20%)	1,717 (52.60%)	106 (43.80%)	202 (54.01%)	0.001
Single-child status, n (%)	5,774 (68.20%)	2,110 (64.64%)	186 (76.86%)	270 (72.19%)	<0.0001
Rural, n (%)	3,837 (45.32%)	1,305 (39.98%)	92 (38.02%)	184 (49.20%)	<0.0001
*Province or city (n, %)*					<0.0001
Hunan	981 (11.59%)	226 (6.92%)	14 (5.79%)	9 (2.41%)	
Ningxia	149 (1.76%)	907 (27.79%)	0 (0.00%)	29 (7.75%)	
Tianjin	1,544 (18.24%)	550 (16.85%)	71 (29.34%)	147 (39.30%)	
Chongqing	1,316 (15.54%)	366 (11.21%)	16 (6.61%)	39 (10.43%)	
Liaoning	1,527 (18.04%)	359 (11.00%)	68 (28.10%)	71 (18.98%)	
Shanghai	1,338 (15.80%)	497 (15.23%)	40 (16.53%)	57 (15.24%)	
Guangzhou	1,611 (19.03%)	359 (11.00%)	33 (13.64%)	22 (5.88%)	
**Neonatal factors**					
*Birth* weight, n (%)					<0.0001
Low birth weight	320 (3.78%)	156 (4.78%)	4 (1.65%)	18 (4.81%)	
Normal birth weight	7,395 (87.35%)	2,853 (87.41%)	190 (78.51%)	305 (81.55%)	
High birth weight	751 (8.87%)	255 (7.81%)	48 (19.83%)	51 (13.64%)	
*Breastfeeding duration, n (%)*					<0.0001
Non-breastfeeding	1,480 (17.48%)	500 (15.32%)	43 (17.77%)	58 (15.51%)	
0–6 months	2,694 (31.82%)	974 (29.84%)	79 (32.64%)	125 (33.42%)	
6–12 months	3,017 (35.64%)	1,259 (38.57%)	67 (27.69%)	111 (29.68%)	
>12 months	1,275 (15.06%)	531 (16.27%)	53 (21.90%)	80 (21.39%)	
**Parental or family factors**					
*Paternal weight status, n (%)*					<0.0001
Normal	4,757 (56.19%)	1,686 (51.65%)	83 (34.30%)	121 (32.35%)	
Overweight	2,851 (33.68%)	1,226 (37.56%)	96 (39.67%)	154 (41.18%)	
Obesity	858 (10.13%)	352 (10.78%)	63 (26.03%)	99 (26.47%)	
*Maternal weight status, n (%)*					<0.0001
Normal	6,718 (79.35%)	2,514 (77.02%)	152 (62.81%)	214 (57.22%)	
Overweight	1,447 (17.09%)	619 (18.96%)	68 (28.10%)	116 (31.02%)	
Obesity	301 (3.56%)	131 (4.01%)	22 (9.09%)	44 (11.76%)	
*Paternal education, n (%)*					<0.0001
Primary school or below	539 (6.37%)	269 (8.24%)	11 (4.55%)	23 (6.15%)	
Secondary or equivalent	5,457 (64.46%)	2,237 (68.54%)	151 (62.40%)	262 (70.05%)	
Junior college or above	2,470 (29.18%)	758 (23.22%)	80 (33.06%)	89 (23.80%)	
*Maternal education, n (%)*					<0.0001
Primary school or below	719 (8.49%)	351 (10.75%)	16 (6.61%)	32 (8.56%)	
Secondary or equivalent	5,421 (64.03%)	2,166 (66.36%)	146 (60.33%)	254 (67.91%)	
Junior college or above	2,326 (27.47%)	747 (22.89%)	80 (33.06%)	88 (23.53%)	
*Monthly household income, n (%)*					<0.0001
<5,000 yuan	2,310 (27.29%)	955 (29.26%)	67 (27.69%)	121 (32.35%)	
5,000–12,000 yuan	5,378 (63.52%)	2,088 (63.97%)	161 (66.53%)	237 (63.37%)	
≥12,000 yuan	778 (9.19%)	221 (6.77%)	14 (5.79%)	16 (4.28%)	
**Lifestyle factors**					
*Fruit consumption, n (%)*					0.08
< 0.75 serving/day	3,012 (35.58%)	1,212 (37.13%)	81 (33.47%)	119 (31.82%)	
0.75–1.5 serving/day	2,984 (35.25%)	1,095 (33.55%)	78 (32.23%)	127 (33.96%)	
≥1.5 serving/day	2,470 (29.18%)	957 (29.32%)	83 (34.30%)	128 (34.22%)	
*Vegetable consumption, n (%)*					0.854
<1 serving/day	2,002 (23.65%)	777 (23.81%)	60 (24.79%)	77 (20.59%)	
1–3 serving/day	4,814 (56.86%)	1,851 (56.71%)	133 (54.96%)	217 (58.02%)	
≥3 serving/day	1,650 (19.49%)	636 (19.49%)	49 (20.25%)	80 (21.39%)	
*SSB consumption, n (%)*					0.001
0 serving/day	3,255 (38.45%)	1,341 (41.08%)	100 (41.32%)	128 (34.22%)	
< 1 serving/day	3,343 (39.49%)	1,159 (35.51%)	91 (37.60%)	138 (36.90%)	
≥ 1 serving/day	1,868 (22.06%)	764 (23.41%)	51 (21.07%)	108 (28.88%)	
*Sedentary time, n (%)*					0.042
≤3 h/day	3,698 (43.68%)	1,507 (46.17%)	96 (39.67%)	148 (39.57%)	
3–7 h/day	924 (10.91%)	362 (11.09%)	32 (13.22%)	43 (11.50%)	
≥7 h/day	3,844 (45.41%)	1,395 (42.74%)	114 (47.11%)	183 (48.93%)	
*Screen time, n (%)*					<0.0001
<1 h/day	4,664 (55.09%)	1,962 (60.11%)	112 (46.28%)	210 (56.15%)	
1–3 h/day	2,662 (31.44%)	868 (26.59%)	87 (35.95%)	111 (29.68%)	
≥3 h/day	1,140 (13.47%)	434 (13.30%)	43 (17.77%)	53 (14.17%)	
*Sleep duration, n (%)*					<0.0001
<7 h	1,422 (16.80%)	638 (19.55%)	34 (14.05%)	80 (21.39%)	
7–9 h	5,279 (62.36%)	2,117 (64.86%)	160 (66.12%)	228 (60.96%)	
≥9 h	1,765 (20.85%)	509 (15.59%)	48 (19.83%)	66 (17.65%)	
*Physical activity, n (%)*					0.065
0 h/day	3,447 (40.72%)	1,287 (39.43%)	88 (36.36%)	140 (37.43%)	
< 0.5 h/day	3,327 (39.30%)	1,304 (39.95%)	109 (45.04%)	150 (40.11%)	
≥ 0.5 h/day	1,692 (19.99%)	673 (20.62%)	45 (18.60%)	84 (22.46%)	

MHNW, metabolically healthy normal weight; MHO, metabolically healthy obesity; MUNW, metabolically unhealthy normal weight; MUO, metabolically unhealthy obesity; BMI, body mass index; SBP, systolic blood pressure; DBP, diastolic blood pressure; TG, triglycerides; HDL-C, high-density lipoprotein cholesterol; SSB, sugar-sweetened beverage.

### Prevalence of metabolic body size phenotype according to three definitions

We compared the prevalence of risk factors among different metabolic body size phenotypes in boys and girls using three definitions, which is summarized in **Table 3**. Based on the CMRF criteria, 72.6%, 22.4%, 2.4%, and 2.6% of subjects were MHNW, MUNW, MHO, and MUO, respectively. However, compared to the CMRF criteria, there was a more metabolically healthy phenotype defined by the MetS component definition (MHNW, 82.8%; MHO, 7.3%) while a more metabolically unhealthy phenotype as defined by the 2018 consensus-based criteria (MUNW, 26.4%; MUO, 3.0%). Based on the 2018 consensus-based criteria, a higher prevalence of MUNW and MUO was detected in boys than girls. Similar trends were also presented in the region-weighted rates. Among metabolically unhealthy groups, the abnormal glucose levels accounted for a smaller proportion, while high BP levels, low levels of HDL-C, and high concentrations of TG were the main components. Undoubtedly, MUO groups had worse cardiometabolic profiles than MUNW groups. Agreement between the 2018 consensus-based criteria and CMRF criteria was considered as substantial agreement (kappa coefficient >0.600, **Supplementary Table 2**), while the two standards are more consistent in evaluating metabolic disorders in obese children.

**Table 3:** Distribution of cardiometabolic risk factors among different metabolic body size phenotypes in each sex, based on three widely used definitions.

Cardiometabolic risk factors	MHNW	MUNW	MHO	MUO
CMRF criteria, n (%)	8,967, 72.6	2,763, 22.4	298, 2.4	318, 2.6
Region-weighted rate (%)	78.9	17.4	2.1	1.7
Boys	4,498, 72.7	1,384, 22.3	146, 2.4	162, 2.6
Region-weighted rate (%)	79.0	17.3	2.1	1.6
BMI ≥95th percentile	–	–	146 (100.0%)	162 (100.0%)
SBP or DBP ≥130/85 mmHg	–	210 (15.2%)	–	70 (43.2%)
Glycemia ≥5.6 mmol/L	–	166 (12.0%)	–	11 (6.8%)
HDL cholesterol <1.03/1.29 mmol/L	–	697 (50.4%)	–	91 (56.2%)
TG ≥1.7 mmol/L	–	615 (44.4%)	–	83 (51.2%)
Girls	4,469, 72.6	1,379, 22.4	152, 2.5	156, 2.5
Region-weighted rate (%)	78.8	17.5	2.1	1.7
BMI ≥95th percentile	–	–	152 (100.0%)	156 (100.0%)
SBP or DBP ≥130/85 mmHg	–	111 (8.1%)	–	39 (25.0%)
Glycemia ≥5.6 mmol/L	–	62 (4.5%)	–	9 (5.8%)
HDL cholesterol <1.03/1.29 mmol/L	–	777 (56.4%)	–	95 (60.9%)
TG ≥1.7 mmol/L	–	705 (51.1%)	–	77 (49.4%)
MetS component definition, n (%)	10,222, 82.8	873, 7.1	896, 7.3	355, 2.9
Region-weighted rate (%)	86.4	5.6	5.9	2.0
Boys	5,129, 82.9	435, 7.0	428, 6.9	198, 3.2
Region-weighted rate (%)	86.8	5.5	5.6	2.1
WC ≥90th percentile	–	–	428 (100.0%)	198 (100.0%)
SBP or DBP >90th percentile	374 (7.3%)	198 (45.5%)	78 (18.2%)	127 (64.1%)
Glycemia ≥5.6 mmol/L	112 (2.2%)	43 (9.9%)	5 (1.2%)	17 (8.6%)
HDL cholesterol ≤1.03 mmol/L	306 (6.0%)	302 (69.4%)	46 (10.8%)	134 (67.7%)
TG ≥1.24 mmol/L	641 (12.5%)	385 (88.5%)	102 (23.8%)	170 (85.9%)
Girls	5,093, 82.7	438, 7.1	468, 7.6	157, 2.6
Region-weighted rate (%)	86.0	5.8	6.3	1.9
WC ≥90th percentile	–	–	468 (100.0%)	157 (100.0%)
SBP or DBP >90th percentile	307 (6.0%)	209 (47.7%)	57 (12.2%)	75 (47.8%)
Glycemia ≥5.6 mmol/L	33 (0.7%)	22 (5.0%)	4 (0.9%)	12 (7.6%)
HDL cholesterol ≤1.03 mmol/L	233 (4.6%)	278 (63.5%)	37 (7.9%)	101 (64.3%)
TG ≥1.24 mmol/L	939 (18.4%)	408 (93.2%)	114 (24.4%)	146 (93.0%)
2018 consensus-based criteria, n (%)	8,466, 68.6	3,264, 26.4	242, 2.0	374, 3.0
Region-weighted rate (%)	73.6	22.7	1.7	2.1
Boys	4,165, 67.3	1,717, 27.7	106, 1.7	202, 3.3
Region-weighted rate (%)	72.5	23.7	1.5	2.2
BMI ≥95th percentile	–	–	106 (100.0%)	202 (100.0%)
SBP or DBP >90th percentile	–	644 (37.51%)	–	133 (65.8%)
Glycemia ≥5.6 mmol/L	–	166 (9.67%)	–	11 (5.5%)
HDL cholesterol ≤1.03 mmol/L	–	697 (40.59%)	–	91 (45.1%)
TG ≥11.7 mmol/L	–	615 (35.82%)	–	83 (41.1%)
Girls	4,301, 69.9	1,547, 25.1	136, 2.2	172, 2.8
Region-weighted rate (%)*	74.7	21.6	1.8	2.0
BMI ≥95th percentile	–	–	136 (100.0%)	172 (100.0%)
SBP or DBP >90th percentile	–	567 (36.7%)	–	81 (47.1%)
Glycemia ≥5.6 mmol/L	–	62 (4.0%)	–	9 (5.2%)
HDL cholesterol ≤1.03 mmol/L	–	568 (36.7%)	–	81 (47.1%)
TG ≥11.7 mmol/L	–	705 (45.6%)	–	77 (44.8%)

MHNW, metabolically healthy normal weight; MHO, metabolically healthy obesity; MUNW, metabolically unhealthy normal weight; MUO, metabolically unhealthy obesity; WC, waist circumference; SBP, systolic blood pressure; DBP, diastolic blood pressure; HDL-C, high-density lipoprotein cholesterol; TG, triglycerides.

*Region-weighted rate was weighted by the national population proportion.

### Associated demographic, neonatal, parental, or family factors of metabolic body size phenotype

The logistic regression analysis included adjustments for age, sex, single-child status, and residence area to identify any association of potential demographic, neonatal, parental, or family factors with metabolic body size phenotype (**Table 4**). According to the 2018 consensus-based criteria, with the MHNW as the reference group, compared to children aged 7–12 years, those aged 13–18 years had higher risks of MUNW (OR = 1.38, 95% CI = 1.27–1.50) after full adjustment. Girls had lower odds of MUNW (OR = 0.85, 95% CI = 0.79–0.92) but a higher likelihood of MHO (OR = 1.31, 95% CI = 1.01–1.69). Compared to MHO, older age was still a risk factor, and female sex was a protective indicator for MUO, as shown in **Supplementary Table 3**. High birth weight (OR = 5.79, 95% CI = 2.07–16.24) and prolonged breastfeeding duration (OR = 1.61, 95% CI = 1.06–2.43) were risk factors for MHO. Apart from this, parental overweight or obesity was positively associated with metabolic disorders or obesity (all *p* < 0.05), and higher paternal or maternal education could decrease the odds of MUNW by 40% (OR = 0.60, 95% CI = 0.50–0.71) and 35% (OR = 0.65, 95% CI = 0.56–0.77), respectively. Also, a monthly household income of ≥12,000 CNY could significantly decrease the odds of MUNW by 36% (OR = 0.64, 95% CI = 0.55–0.76).

**Table 4:** Odds ratios for different metabolic obesity phenotypes associated with demographic, neonatal, parental, or family factors, based on 2018 consensus-based criteria.

Characteristics	MUNW*		MHO*		MUO*	
	UnadjustedOR (95% CI)	ModelOR (95% CI)	UnadjustedOR (95% CI)	ModelOR (95% CI)	UnadjustedOR (95% CI)	ModelOR (95% CI)
**Demographic factors**						
*Age*						
7–12 years	1 (Reference)	1 (Reference)	1 (Reference)	1 (Reference)	1 (Reference)	1 (Reference)
13–18 years	**1.31 (1.20–1.42)**	**1.38 (1.27–1.50)**	0.79 (0.60–1.04)	0.81 (0.61–1.08)	**1.31 (1.06–1.61)**	**1.29 (1.04–1.60)**
*Sex*						
Boy	1 (Reference)	1 (Reference)	1 (Reference)	1 (Reference)	1 (Reference)	1 (Reference)
Girl	**0.87 (0.81–0.95)**	**0.85 (0.79–0.92)**	1.24 (0.96–1.61)	**1.31 (1.01–1.69)**	0.83 (0.67–1.02)	**0.82 (0.67–0.99)**
*Single-child status*						
No	1 (Reference)	1 (Reference)	1 (Reference)	1 (Reference)	1 (Reference)	1 (Reference)
Yes	**0.85 (0.78–0.93)**	**0.83 (0.76–0.91)**	**1.55 (1.15–2.10)**	**1.58 (1.17–2.14)**	1.21 (0.96–1.53)	1.18 (0.94–1.49)
*Residence area*						
Rural	1 (Reference)	1 (Reference)	1 (Reference)	1 (Reference)	1 (Reference)	1 (Reference)
Urban	**0.80 (0.74–0.87)**	**0.75 (0.69–0.82)**	**1.35 (1.04–1.76)**	1.24 (0.95–1.63)	1.05 (0.87–1.26)	1.00 (0.82–1.21)
**Neonatal factors**						
*Birth weight, n (%)*						
Low birth weight	1 (Reference)	1 (Reference)	1 (Reference)	1 (Reference)	1 (Reference)	1 (Reference)
Normal birth weight	**0.79 (0.65–0.96)**	**0.80 (0.66–0.97)**	2.06 (0.76–5.57)	2.16 (0.80–5.85)	0.73 (0.45–1.20)	0.68 (0.42–1.12)
High birth weight	**0.70 (0.55–0.88)**	**0.67 (0.52–0.85)**	**5.11 (1.83–14.30)**	**5.79 (2.07–16.24)**	1.21 (0.69–2.10)	1.13 (0.65–1.97)
*Breastfeeding duration, n (%)*						
Non-breastfeeding	1 (Reference)	1 (Reference)	1 (Reference)	1 (Reference)	1 (Reference)	1 (Reference)
0–6 months	1.07 (0.94–1.21)	1.08 (0.95–1.22)	1.01 (0.69–1.47)	1.03 (0.71–1.50)	1.18 (0.86–1.63)	1.21 (0.88–1.66)
6–12 months	**1.24 (1.10–1.39)**	**1.24 (1.10–1.41)**	0.76 (0.52–1.13)	0.81 (0.55–1.19)	0.94 (0.68–1.30)	0.97 (0.70–1.34)
>12 months	**1.23 (1.07–1.42)**	**1.29 (1.12–1.50)**	1.43 (0.95–2.15)	**1.61 (1.06–2.43)**	**1.60 (1.13–2.26)**	**1.67 (1.18–2.38)**
**Parental or family factors**						
*Paternal weight status*						
Normal	1 (Reference)	1 (Reference)	1 (Reference)	1 (Reference)	1 (Reference)	1 (Reference)
Overweight	**1.21 (1.11–1.32)**	**1.21 (1.11–1.32)**	**1.93 (1.43–2.60)**	**1.91 (1.42–2.57)**	**2.12 (1.67–2.71)**	**2.16 (1.70–2.76)**
Obesity	**1.16 (1.01–1.33)**	**1.17 (1.02–1.34)**	**4.21 (3.01–5.89)**	**4.06 (2.90–5.69)**	**4.54 (3.44–5.97)**	**4.76 (3.61–6.28)**
*Maternal weight status*						
Normal	1 (Reference)	1 (Reference)	1 (Reference)	1 (Reference)	1 (Reference)	1 (Reference)
Overweight	**1.14 (1.03–1.27)**	**1.13 (1.01–1.25)**	**2.08 (1.55–2.78)**	**2.24 (1.67–3.01)**	**2.52 (1.99–3.18)**	**2.59 (2.04–3.27)**
Obesity	1.16 (0.94–1.44)	1.17 (0.94–1.44)	**3.23 (2.04–5.13)**	**3.52 (2.21–5.60)**	**4.59 (3.25–6.48)**	**4.71 (3.33–6.66)**
*Paternal educational attainment*						
Primary school or below	1 (Reference)	1 (Reference)	1 (Reference)	1 (Reference)	1 (Reference)	1 (Reference)
Secondary or equivalent	**0.81 (0.70–0.95)**	**0.80 (0.68–0.94)**	1.41 (0.76–2.62)	1.22 (0.66–2.28)	1.12 (0.73–1.74)	1.09 (0.70–1.70)
Junior college or above	**0.62 (0.52–0.73)**	**0.60 (0.50–0.71)**	1.59 (0.84–3.00)	1.12 (0.58–2.16)	0.85 (0.53–1.35)	0.82 (0.50–1.33)
*Maternal educational attainment*						
Primary school or below	1 (Reference)	1 (Reference)	1 (Reference)	1 (Reference)	1 (Reference)	1 (Reference)
Secondary or equivalent	**0.81 (0.71–0.93)**	**0.81 (0.70–0.93)**	1.27 (0.76–2.15)	1.07 (0.63–1.82)	1.04 (0.72–1.52)	1.01 (0.68–1.48)
Junior college or above	**0.66 (0.57–0.77)**	**0.65 (0.56–0.77)**	1.55 (0.90–2.66)	1.06 (0.60–1.88)	0.85 (0.56–1.29)	0.81 (0.53–1.26)
*Monthly household income*						
<5,000 yuan	1 (Reference)	1 (Reference)	1 (Reference)	1 (Reference)	1 (Reference)	1 (Reference)
5,000–12,000 yuan	**0.81 (0.74–0.89)**	**0.82 (0.75–0.90)**	1.12 (0.84–1.51)	1.05 (0.78–1.42)	0.79 (0.63–1.01)	**0.80 (0.62–0.99)**
≥12,000 yuan	**0.63 (0.54–0.75)**	**0.64 (0.55–0.76)**	0.62 (0.35–1.11)	**0.53 (0.30–0.95)**	**0.39 (0.23–0.67)**	**0.41 (0.24–0.69)**

Model: adjusted for age, sex, single-child status, and residence area. Bold values refer to p < 0.05.

MUNW, metabolically unhealthy with normal weight; MHO, metabolically healthy obesity; MUO, metabolically unhealthy obesity.

*MHNW was regarded as the reference group, and the metabolic obesity phenotype was defined by 2018 consensus-based criteria.

Not surprisingly, based on the CMRF criteria and MetS component criteria, younger age, single-child status, urban residence, high birth weight, prolonged breastfeeding duration, and parental overweight/obesity status were still important predictors for MHO (**Supplementary Tables 4**, **5**).

### Associated lifestyle factors of metabolic body size phenotype

The adjusted ORs for lifestyle factors in relation to metabolic body size phenotype defined by the 2018 consensus-based criteria are summarized in **Table 5**. Regarding the MHNW as the reference group, a prolonged screen time ≥3 h/day was positively correlated with MHO (OR = 1.83, 95% CI = 1.27–2.63); however, doing exercise ≥0.5 h/day could decrease the odds of MHO by 39% (OR = 0.61, 95% CI = 0.42–0.89) and MUNW by 11% (OR = 0.89, 95% CI = 0.80–0.98). When MHO was used as a reference, SSB over-consumption, inadequate sleep duration, and less physical activity remained risk factors for MUO (**Supplementary Table 6**).

**Table 5:** Odds ratios for different metabolic obesity phenotypes associated with lifestyle factors, based on 2018 consensus-based criteria.

Characteristics	MUNW*		MHO*		MUO*	
	UnadjustedOR (95% CI)	ModelOR (95% CI)	UnadjustedOR (95% CI)	ModelOR (95% CI)	UnadjustedOR (95% CI)	ModelOR (95% CI)
**Lifestyle factors**						
*Fruit consumption*						
< 0.75 serving/day	1 (Reference)	1 (Reference)	1 (Reference)	1 (Reference)	1 (Reference)	1 (Reference)
0.75–1.5 serving/day	0.91 (0.83–1.00)	0.94 (0.85–1.04)	0.97 (0.71–1.33)	0.95 (0.69–1.31)	1.08 (0.84–1.39)	1.10 (0.85–1.42)
≥1.5 serving/day	0.96 (0.87–1.06)	0.98 (0.88–1.08)	1.25 (0.92–1.71)	1.20 (0.88–1.64)	**1.31 (1.02–1.69)**	**1.35 (1.04–1.74)**
*Vegetable consumption*						
<1 serving/day	1 (Reference)	1 (Reference)	1 (Reference)	1 (Reference)	1 (Reference)	1 (Reference)
1–3 serving/day	0.99 (0.90–1.09)	0.99 (0.90–1.09)	0.92 (0.68–1.26)	0.90 (0.66–1.23)	1.17 (0.90–1.53)	1.17 (0.90–1.53)
≥3 serving/day	0.99 (0.88–1.12)	0.97 (0.85–1.09)	0.99 (0.68–1.45)	0.99 (0.67–1.45)	1.26 (0.92–1.74)	1.24 (0.90–1.71)
*SSB consumption*						
0 serving/day	1 (Reference)	1 (Reference)	1 (Reference)	1 (Reference)	1 (Reference)	1 (Reference)
<1 serving/day	**0.84 (0.77–0.92)**	**0.84 (0.76–0.92)**	0.89 (0.66–1.18)	0.90 (0.67–1.20)	1.05 (0.82–1.34)	1.04 (0.82–1.34)
≥1 serving/day	0.99 (0.89–1.10)	0.91 (0.82–1.02)	0.89 (0.63–1.25)	1.03 (0.73–1.47)	**1.47 (1.13–1.91)**	**1.31 (1.01–1.72)**
*Sedentary time*						
≤3 h/day	1 (Reference)	1 (Reference)	1 (Reference)	1 (Reference)	1 (Reference)	1 (Reference)
3–7 h/day	1.08 (0.98–1.20)	1.07 (0.96–1.18)	1.33 (0.89–2.00)	1.30 (0.86–1.95)	1.16 (0.82–1.65)	1.16 (0.82–1.64)
≥7 h/day	1.07 (0.97–1.17)	0.92 (0.84–1.02)	1.14 (0.87–1.50)	1.16 (0.87–1.53)	1.19 (0.95–1.48)	1.12 (0.89–1.40)
*Screen time*						
<1 h/day	1 (Reference)	1 (Reference)	1 (Reference)	1 (Reference)	1 (Reference)	1 (Reference)
1–3 h/day	**0.86 (0.79–0.93)**	0.93 (0.85–1.01)	**1.36 (1.02–1.81)**	**1.41 (1.06–1.87)**	0.93 (0.73–1.17)	0.94 (0.74–1.19)
≥3 h/day	1.05 (0.94–1.17)	0.95 (0.84–1.06)	**1.57 (1.10–2.25)**	**1.83 (1.27–2.63)**	1.03 (0.76–1.41)	0.98 (0.72–1.34)
*Sleep duration*						
<7 h	1 (Reference)	1 (Reference)	1 (Reference)	1 (Reference)	1 (Reference)	1 (Reference)
7–9 h	**0.86 (0.77–0.96)**	0.92 (0.82–1.03)	1.32 (0.91–1.93)	1.14 (0.77–1.70)	**0.76 (0.58–0.99)**	0.83 (0.62–1.10)
≥9 h	**0.64 (0.56–0.74)**	**0.73 (0.63–0.85)**	1.14 (0.73–1.77)	0.89 (0.55–1.45)	**0.66 (0.48–0.93)**	0.78 (0.54–1.14)
*Physical activity*						
0 h/day	1 (Reference)	1 (Reference)	1 (Reference)	1 (Reference)	1 (Reference)	1 (Reference)
<0.5 h/day	**0.91 (0.83–0.99)**	**0.88 (0.80–0.96)**	0.78 (0.57–1.07)	0.78 (0.57–1.07)	0.88 (0.70–1.10)	0.82 (0.65–1.02)
≥0.5 h/day	0.93 (0.84–1.03)	**0.89 (0.80–0.98)**	**0.59 (0.40–0.86)**	**0.61 (0.42–0.89)**	0.99 (0.78–1.27)	0.93 (0.73–1.19)

Model: adjusted for age, sex, single-child status, and residence area. Bold values refer to p < 0.05.

MUNW, metabolically unhealthy with normal weight; MHO, metabolically healthy obesity; MUO, metabolically unhealthy obesity.

*MHNW was regarded as the reference group, and the metabolic obesity phenotype was defined by 2018 consensus-based criteria.

Consistent with the main results, when we redefined the outcomes with reference to the CMRF criteria and MetS component criteria, prolonged screen time and inadequate physical activity were still independent modifiable predictors for MHO (**Supplementary Tables 7**, **8**).

## Discussion

To our knowledge, this is the first study from a national level to characterize and compare the prevalence and further investigate the inherent and modifiable predictors of metabolic body size phenotype in the Chinese pediatric population. Compared to the CMRF criteria and MetS component definition, the application of the 2018 consensus-based definition aims to find more metabolic abnormalities in both normal weight or obese children, whereas the 2018 consensus-based definition and CMRF criteria are less different for the diagnosis of metabolic abnormalities or obesity in children. Factors positively associated with MHO were younger age, single-child status, urban residence, high birth weight, prolonged breastfeeding duration, parental overweight/obesity status, long screen time, and less physical activity. Older age, male sex, rural residence, more consumption of SSB, inadequate sleep duration, and physical activity might contribute to MUO with reference to MHO. Taken together, the study’s findings provide additional insights on intervention strategies aimed at improving metabolic or weight health in children and adolescents.

According to the 2018 consensus-based criteria that hold potential universal value to enable comparisons between studies and inform clinical decision-making for children with obesity (22), a higher rate of 47.6% MHO phenotype was observed among 5–16-year obese Greek children and adolescents (38); however, it must be considered that this sample did not originate from a population screening. The Asian population showed a lower rate of MHO than Western people; this discrepancy could be due to different fat distribution by ethnicity and the influence of genetic, cultural, or environmental factors. However, Genovesi and his colleagues proposed that the consensus criteria were a bit limiting since they did not cover all potential cardiovascular risk factors, such as insulin resistance and high levels of uric acid (39). Despite the limitation raised, we have to acknowledge that the consensus-based definition might identify more metabolically unhealthy individuals since it defines MUO as obesity without any cardiometabolic factors rather than with less than two potential risk factors, and the criteria of high BP takes into account age and sex differences and are therefore more precise than just considering as whether it is greater than 130/85 mmHg. From this perspective, the 2018 consensus-based definition has critical implications for obesity management, health system resource allocation, and clinical research.

Demographic factors were considered important predictors, with some studies reporting a higher incidence of metabolically healthy phenotype among girls and younger adolescents (40, 41). Similar results were observed in the multi-ethnic Asian cohort (42). Specifically, the risks of MUNW or MUO brought by older ages could be explained that visceral fat tissue accumulates faster with age, eventually leading to metabolic abnormalities (43). Furthermore, girls usually have lower fat levels in the visceral deposits, as a result of diverse sexual maturation and physical fitness in girls, which seems to confer a lower level of systemic metabolic risk (44). In addition, the prevalence of metabolic body size phenotype was different according to single-child status, residence area, parental weight status, parental education, and monthly household income. As a risk predictor of MHO, being a single child may be associated with a higher risk of elevated BP and abdominal obesity (45, 46). The single child may be overfed and indulged in the whole family, while nutrition may lead to these being converted into weight gain. High socioeconomic status in an urban area with financial freedom also allows better access to quality nutrition that encourages healthy lifestyle behaviors. Furthermore, despite genetic predisposition, parents and their children are often exposed to similar environments and share similar lifestyles, and a higher parental education level might be related to greater availability of healthy foods. Therefore, parental overweight/obesity or educational attainment was identified as a strong risk factor for obesity or metabolic unhealthy phenotype in their offspring. As the home environment can influence the lifestyle habits of children, successful home-based interventions should take into account parents’ beliefs and intentions, and work with parents to positively reconcile differences in these beliefs and intentions with the day-to-day difficulties and pressures faced.

Lifestyle behaviors were crucial for children’s health. Screen time and physical activities were associated with MHO in children and adolescents. TV or computer viewing was associated with obesity, and it may be an indicator of sedentary behavior (47). It has been suggested that screen viewing has a lowering effect on the metabolic rate in children, but the data are not conclusive (48). Reducing screen viewing is likely to prevent weight gain either directly or indirectly. Furthermore, regular exercise is effective in preventing obesity, and a lower level of physical activity is an important predictor of the MHO phenotype (49), but compared to MUO, MHO individuals were more active and spent less time in sedentary behaviors (50).

Over-consumption of SSBs, inadequate sleep duration, or physical activities might contribute to the development of MUO, compared to MHO. The hazards of sweet beverages are widely confirmed, and the associations between SSB and MetS had been extensively assessed in observational studies in Chinese children and adolescents (51, 52). Sugar could induce a fast increase in blood glucose and may lead to oxidative stress, as a consequence of which vascular damage and metabolic disorders (53). Apart from this, inadequate sleep was a potential predictor of metabolic abnormalities (54); a possible explanation is that short sleep duration decreases nocturnal leptin production and increases ghrelin with a net effect of increasing appetite and fatigue, which in turn leads to higher caloric intake (55). In addition, it is biologically plausible that physical activity improves the metabolic-risk profile independent of adiposity, such as improving insulin action and glucose transport (56). Familiarity with the prevalence as well as the associated factors of metabolic abnormalities is helpful in planning preventive measures.

Based on widely used criteria (22, 23), considering the lack of data with Chinese national samples on the prevalence of these metabolic body size phenotypes and associated factors, the present study brings important contributions to this theme. Since individuals with MHO are at increased risk of cardiovascular disease, we propose that educational programs should consider these findings and be implemented widely to make the public aware of the importance of healthy lifestyles, especially in the high-risk population. Our findings may have important implications for developing public health policies and effective intervention programs. For the different stages of obesity management (**Supplementary Figure 1**), it is recommended that children and adolescents spend as little time as possible engaging in electronic screen activity to avoid the parallel increase in sedentary times. Also, regular exercise is effective in preventing obesity. To avoid the progression of MHO towards MUO, sugar-related diet control could be a potential target intervention among children and adolescents. School health teams should offer children and adolescents a soft drink alternative by providing access to healthy drinks and encouraging students to engage in more outdoor activities. Apart from this, children and adolescents are recommended to have an adequate duration and high quality of sleep to prevent the occurrence of metabolic disorders. Meanwhile, effective social media reaching the younger population should be applied to make them aware of the potentially harmful consequences of sugar, inadequate physical activities, and less sleep duration. In addition, introduced by the Chinese government and conducted as a social or political issue, China’s “double reduction policy” aims to ease the burden of excessive homework and off-campus tutoring for young students, which can serve the purpose of supporting healthy behaviors and promoting physical wellbeing, which needs to be vigorously promoted and implemented.

Our previous finding suggested that a continued increase in fruit consumption would result in childhood bad lipid health (30). In the present study, we further detected several characteristics for the stratification management of different metabolic body size phenotypes in children and adolescents, and we determined the associated lifestyle factors in the different stages of intervention, in addition to the comparisons of three widely used MHO definitions. The strength of the study is the large sample size of the study population recruited in schools from seven provinces of China, which might be nationally representative. Anthropometric and blood pressure measurements were obtained using standardized protocols rather than being self-reported. Notwithstanding, we adopted the timely updated consensus-based definition and compared it with other widely used criteria to define MHO in the pediatric population. However, several limitations should be paid attention to when interpreting the findings of other populations. First, since the majority of the study population was of Han ethnicity, our results may not be applicable to other ethnic groups. Second, the habitual lifestyle factors including dietary habits were obtained from self-reported surveys, and there was a possibility of recall bias. However, child questionnaires of children grades 1–3 were also reported by parents, and trained project members interpreted all the questionnaires in detail, and also they would give appropriate guidance as effectively as possible. The questionnaires would be rechecked by 3% within 1 week for the same participants. Therefore, the quality of self-reported information was largely guaranteed. Finally, the present study was a cross-sectional design. For this reason, we can only describe associations between potential factors and the presence of different metabolic body size phenotypes, but we cannot say whether or not there is a cause/effect relationship.

## Conclusion

Compared to the CMRF criteria and MetS component definition, the application of the 2018 consensus-based definition aims to find more metabolic abnormalities in both normal weight and obese children, and it reaches a substantial agreement with the CMRF criteria. Younger age, single-child status, and those who came from the urban area are high-risk populations to develop metabolically healthy obesity, while older-aged children, boys, and those who came from the rural area might be an important target population for preventing MUO. Findings may be used in the development of intervention strategies to promote parental educational programs and healthy lifestyle initiatives aimed at improving metabolic or weight health in children and adolescents.

## Data availability statement

The raw data supporting the conclusions of this article will be made available by the authors, without undue reservation.

## Ethics statement

The studies involving human participants were reviewed and approved by the Ethics Committee of Peking University (Number: IRB0000105213034). Written informed consent to participate in this study was provided by the participants’ legal guardian/next of kin.

## Author contributions

Conceptualization: JL Data curation: TM, MC, and YM Formal analysis: JL Funding acquisition: YD and JM Methodology: JL, TM, YL, DG, and QM Project administration: YD and JM Resources: YD and JM Software: JL, QM, XW, and LC Supervision: YD and YS Validation: YD and YS Visualization: TM, MC, YM, YL, DG, and YZ Writing—original draft: JL Writing—review and editing: TM, MC, YM, YL, DG, JM, YD, and YS. All authors have read and agreed to the published version of the manuscript.

## Funding

This research was funded by the China Postdoctoral Science Foundation (BX20200019 and 2020M680266 to YD), National Natural Science Foundation of China (82103865 to YD), and Beijing Natural Science Foundation (7222244 to YD and 7222247 to YS).

## Acknowledgments

The authors would like to acknowledge the support from all the team members and the participating students, teachers, parents, and local education and health staff in the programs.

## Conflict of interest

The authors declare that the research was conducted in the absence of any commercial or financial relationships that could be construed as a potential conflict of interest.

## Publisher’s note

All claims expressed in this article are solely those of the authors and do not necessarily represent those of their affiliated organizations, or those of the publisher, the editors and the reviewers. Any product that may be evaluated in this article, or claim that may be made by its manufacturer, is not guaranteed or endorsed by the publisher.
