# Supplementary material for: Prevalence and associated factors of metabolic body size phenotype in children and adolescents: A national cross-sectional analysis in China
Source: Front Endocrinol (Lausanne). 2022 Aug 25;13:952825. doi: 10.3389/fendo.2022.952825 (PMC9452664; doi:10.3389/fendo.2022.952825)
Supplement: Supplementary file 1 [file DataSheet_1.docx]

**Supplementary file**

| Supplementary Table 1. The population proportion in each city or province based on 2020 national census data. | | | | | | |
| --- | --- | --- | --- | --- | --- | --- |
|  | 7-18 years aged Boys | Proportion (%) | 7-18 years aged Girls | Proportion (%) | Total | Proportion (%) |
| Hunan | 4750319 | 0.38 | 4127178 | 0.37 | 8877497 | 0.38 |
| Ningxia | 560745 | 0.05 | 536447 | 0.05 | 1097192 | 0.05 |
| Tianjin | 743726 | 0.06 | 640361 | 0.06 | 1384087 | 0.06 |
| Chongqing | 2351505 | 0.19 | 2178731 | 0.20 | 4530235 | 0.19 |
| Liaoning | 2079452 | 0.17 | 1924770 | 0.17 | 4004222 | 0.17 |
| Shanghai | 960114 | 0.08 | 875888 | 0.08 | 1836001 | 0.08 |
| Guangzhou | 937770 | 0.08 | 803423 | 0.07 | 1741193 | 0.07 |
| Total | 12383630 | - | 11086797 | - | 23470427 | - |

| Supplementary Table 2. Kappa tests of different metabolic body size phenotype defined by three definitions in each sex. | | | | |
| --- | --- | --- | --- | --- |
| MetS components definition | MHNW | MUNW | MHO | MUO |
| **CMRFs criteria, n(%)** | | | | |
| **Total population** |  |  |  |  |
| MHNW | 0.486, P<0.0001 | - | - | - |
| MUNW | - | 0.359, P<0.0001 | - | - |
| MHO | - | - | 0.406, P<0.0001 | - |
| MUO | - | - | - | 0.556, P<0.0001 |
| **Boys** |  |  |  |  |
| MHNW | 0.510, P<0.0001 | - | - | - |
| MUNW | - | 0.364, P<0.0001 | - | - |
| MHO | - | - | 0.426, P<0.0001 | - |
| MUO | - | - | - | 0.537, P<0.0001 |
| **Girls** |  |  |  |  |
| MHNW | 0.463, P<0.0001 | - | - | - |
| MUNW | - | 0.354, P<0.0001 | - | - |
| MHO | - | - | 0.387, P<0.0001 | - |
| MUO | - | - | - | 0.577, P<0.0001 |
| **2018 consensus-based criteria, n(%)** | | | | |
| **Total population** |  |  |  |  |
| MHNW | 0.468, P<0.0001 | - | - | - |
| MUNW | - | 0.342, P<0.0001 | - | - |
| MHO | - | - | 0.351, P<0.0001 | - |
| MUO | - | - | - | 0.546, P<0.0001 |
| **Boys** |  |  |  |  |
| MHNW | 0.465, P<0.0001 | - | - | - |
| MUNW | - | 0.321, P<0.0001 | - | - |
| MHO | - | - | 0.341, P<0.0001 | - |
| MUO | - | - | - | 0.514, P<0.0001 |
| **Girls** |  |  |  |  |
| MHNW | 0.472, P<0.0001 | - | - | - |
| MUNW | - | 0.365, P<0.0001 | - | - |
| MHO | - | - | 0.359, P<0.0001 | - |
| MUO | - | - | - | 0.585, P<0.0001 |
| CMRFs criteria | MHNW | MUNW | MHO | MUO |
| **2018 consensus-based criteria, n(%)** | | | | |
| **Total population** |  |  |  |  |
| MHNW | 0.835, P<0.0001 | - | - | - |
| MUNW | - | 0.815, P<0.0001 | - | - |
| MHO | - | - | 0.860, P<0.0001 | - |
| MUO | - | - | - | 0.890, P<0.0001 |
| **Boys** |  |  |  |  |
| MHNW | 0.869, P<0.0001 | - | - | - |
| MUNW | - | 0.854, P<0.0001 | - | - |
| MHO | - | - | 0.838, P<0.0001 | - |
| MUO | - | - | - | 0.887, P<0.0001 |
| **Girls** |  |  |  |  |
| MHNW | 0.800. P<0.0001 | - | - | - |
| MUNW | - | 0.774, P<0.0001 | - | - |
| MHO | - | - | 0.879, P<0.0001 | - |
| MUO | - | - | - | 0.894, P<0.0001 |

| Supplementary Table 3. Odds ratios for MUO associated with demographic, neonatal, parental or family factors, with the reference of MHO. | | | | | | |
| --- | --- | --- | --- | --- | --- | --- |
| Characteristics | 2018 consensus-based criteria-MUO* | | CMRFs criteria-MUO* | | MetS components criteria-MUO* | |
|  | Unadjusted | Model | Unadjusted | Model | Unadjusted | Model |
|  | OR (95% CI) | OR (95% CI) | OR (95% CI) | OR (95% CI) | OR (95% CI) | OR (95% CI) |
|  |  |  |  |  |  |  |
| **Demographic factors** |  |  |  |  |  |  |
| *Age* |  |  |  |  |  |  |
| 7-12 years | 1 (Reference) | 1 (Reference) | 1 (Reference) | 1 (Reference) | 1 (Reference) | 1 (Reference) |
| 13-18 years | **1.66 (1.18-2.34)** | **1.57 (1.10-2.24)** | **3.18 (2.25-4.49)** | **3.03 (2.13-4.32)** | **1.54 (1.20-1.97)** | **1.52 (1.18-1.97)** |
| *Sex* |  |  |  |  |  |  |
| Boy | 1 (Reference) | 1 (Reference) | 1 (Reference) | 1 (Reference) | 1 (Reference) | 1 (Reference) |
| Girl | **0.66 (0.48-0.92)** | **0.61 (0.44-0.86)** | 0.93 (0.67-1.27) | 0.84 (0.60-1.18) | **0.73 (0.57-0.93)** | **0.70 (0.54-0.90)** |
| *Single-child status* |  |  |  |  |  |  |
| No | 1 (Reference) | 1 (Reference) | 1 (Reference) | 1 (Reference) | 1 (Reference) | 1 (Reference) |
| Yes | 0.78 (0.54-1.14) | 0.71 (0.48-1.04) | 0.78 (0.54-1.12) | **0.68 (0.46-0.99)** | 0.96 (0.73-1.26) | 0.87 (0.66-1.16) |
| *Residence area* |  |  |  |  |  |  |
| Rural | 1 (Reference) | 1 (Reference) | 1 (Reference) | 1 (Reference) | 1 (Reference) | 1 (Reference) |
| Urban | **0.63 (0.46-0.88)** | 0.71 (0.51-1.01) | **0.58 (0.42-0.80)** | 0.74 (0.53-1.05) | 0.83 (0.65-1.06) | 0.97 (0.75-1.25) |
| **Neonatal factors** |  |  |  |  |  |  |
| *Birthweight, n(%)* |  |  |  |  |  |  |
| Low birthweight | 1 (Reference) | 1 (Reference) | 1 (Reference) | 1 (Reference) | 1 (Reference) | 1 (Reference) |
| Normal birthweight | 0.36 (0.12-1.07) | 0.37 (0.12-1.12) | 0.63 (0.26-1.52) | 0.69 (0.27-1.80) | 0.87 (0.43-1.75) | 0.87 (0.42-1.77) |
| High birthweight | **0.24 (0.08-0.75)** | **0.23 (0.07-0.74)** | 0.48 (0.18-1.24) | 0.47 (0.17-1.32) | 0.56 (0.26-1.21) | 0.55 (0.25-1.20) |
| *Breastfeeding duration, n(%)* |  |  |  |  |  |  |
| Non-breastfeeding | 1 (Reference) | 1 (Reference) | 1 (Reference) | 1 (Reference) | 1 (Reference) | 1 (Reference) |
| 0-6 months | 1.17 (0.72-1.91) | 1.14 (0.69-1.87) | 0.94 (0.58-1.52) | 0.87 (0.52-1.46) | 0.87 (0.60-1.25) | 0.86 (0.59-1.25) |
| 6-12 months | 1.23 (0.75-2.02) | 1.25 (0.75-2.09) | 1.02 (0.62-1.66) | 1.00 (0.60-1.69) | 0.92 (0.64-1.33) | 0.94 (0.65-1.36) |
| ＞12 months | 1.12 (0.66-1.89) | 1.10 (0.64-1.90) | 0.76 (0.45-1.28) | 0.77 (0.44-1.34) | 0.92 (0.61-1.38) | 0.93 (0.62-1.41) |
| **Parental or family factors** |  |  |  |  |  |  |
| *Paternal weight status* |  |  |  |  |  |  |
| Normal | 1 (Reference) | 1 (Reference) | 1 (Reference) | 1 (Reference) | 1 (Reference) | 1 (Reference) |
| Overweight | 1.10 (0.75-1.61) | 1.13 (0.76-1.67) | 1.06 (0.73-1.54) | 1.06 (0.71-1.57) | 1.08 (0.82-1.43) | 1.05 (0.80-1.39) |
| Obesity | 1.08 (0.71-1.64) | 1.24 (0.80-1.91) | 0.80 (0.53-1.21) | 0.97 (0.62-1.51) | 0.98 (0.70-1.37) | 1.04 (0.74-1.46) |
| *Maternal weight status* |  |  |  |  |  |  |
| Normal | 1 (Reference) | 1 (Reference) | 1 (Reference) | 1 (Reference) | 1 (Reference) | 1 (Reference) |
| Overweight | 1.21 (0.84-1.74) | 1.23 (0.84-1.79) | 1.13 (0.79-1.61) | 1.14 (0.78-1.67) | 1.14 (0.86-1.52) | 1.14 (0.86-1.52) |
| Obesity | 1.42 (0.82-2.47) | 1.47 (0.83-2.61) | 1.19 (0.70-2.01) | 1.14 (0.78-1.67) | 0.98 (0.63-1.55) | 0.96 (0.60-1.52) |
| *Paternal educational attainment* |  |  |  |  |  |  |
| Primary school or below | 1 (Reference) | 1 (Reference) | 1 (Reference) | 1 (Reference) | 1 (Reference) | 1 (Reference) |
| Secondary or equivalent | 0.80 (0.38-1.68) | 0.86 (0.40-1.85) | 0.90 (0.45-1.82) | 1.10 (0.52-2.33) | 0.87 (0.49-1.54) | 0.89 (0.50-1.58) |
| Junior college or above | 0.53 (0.24-1.16) | 0.68 (0.30-1.54) | 0.65 (0.31-1.36) | 1.00 (0.45-2.22) | 0.65 (0.36-1.18) | 0.69 (0.37-1.28) |
| *Maternal educational attainment* |  |  |  |  |  |  |
| Primary school or below | 1 (Reference) | 1 (Reference) | 1 (Reference) | 1 (Reference) | 1 (Reference) | 1 (Reference) |
| Secondary or equivalent | 0.82 (0.43-1.55) | 0.85 (0.44-1.63) | 1.11 (0.61-2.03) | 1.28 (0.67-2.44) | 1.25 (0.77-2.02) | 1.25 (0.77-2.06) |
| Junior college or above | 0.55 (0.28-1.08) | 0.67 (0.33-1.37) | 0.67 (0.35-1.28) | 0.91 (0.45-1.85) | 0.87 (0.52-1.46) | 0.91 (0.52-1.57) |
| *Monthly household income* |  |  |  |  |  |  |
| < 5000 yuan | 1 (Reference) | 1 (Reference) | 1 (Reference) | 1 (Reference) | 1 (Reference) | 1 (Reference) |
| 5000-12000 yuan | 0.71 (0.49-1.03) | 0.70 (0.48-1.03) | 0.84 (0.59-1.21) | 0.85 (0.58-1.25) | 0.93 (0.70-1.23) | 0.94 (0.70-1.25) |
| ≥12000 yuan | 0.63 (0.29-1.38) | 0.54 (0.24-1.20) | 1.03 (0.48-2.22) | 0.85 (0.37-1.95) | **0.55 (0.31-0.98)** | 0.56 (0.32-1.01) |
| Model: adjusted for age, sex, single-child status and residence area. | | | | | | |
| *MHO was regarded as the reference group. | | | | | | |
| Bold values referred to P <0.05. | | | | | | |

| Supplementary Table 4. Odds ratios for different metabolic body size phenotype associated with demographic, neonatal, parental or family factors, based on CMRFs criteria. | | | | | | |
| --- | --- | --- | --- | --- | --- | --- |
| Characteristics | MUNW* | | MHO* | | MUO* | |
|  | Unadjusted OR (95% CI) | Model OR (95% CI) | Unadjusted OR (95% CI) | Model OR (95% CI) | Unadjusted OR (95% CI) | Model OR (95% CI) |
|  |  |  |  |  |  |  |
| **Demographic factors** |  |  |  |  |  |  |
| *Age* |  |  |  |  |  |  |
| 7-12 years | 1 (Reference) | 1 (Reference) | 1 (Reference) | 1 (Reference) | 1 (Reference) | 1 (Reference) |
| 13-18 years | **2.07 (1.90-2.25)** | **2.28 (2.08-2.49)** | **0.63 (0.48-0.83)** | **0.66 (0.50-0.87)** | **2.01 (1.61-2.51)** | **1.98 (1.57-2.48)** |
| *Sex* |  |  |  |  |  |  |
| Boy | 1 (Reference) | 1 (Reference) | 1 (Reference) | 1 (Reference) | 1 (Reference) | 1 (Reference) |
| Girl | 1.00 (0.92-1.09) | 0.96 (0.88-1.05) | 1.05 (0.83-1.32) | 1.09 (0.87-1.38) | 0.97 (0.78-1.21) | 0.96 (0.76-1.20) |
| *Single-child status* |  |  |  |  |  |  |
| No | 1 (Reference) | 1 (Reference) | 1 (Reference) | 1 (Reference) | 1 (Reference) | 1 (Reference) |
| Yes | **0.82 (0.75-0.90)** | **0.79 (0.72-0.87)** | **1.51 (1.15-1.99)** | **1.52 (1.16-2.00)** | 1.18 (0.92-1.51) | 1.15 (0.89-1.47) |
| *Residence area* |  |  |  |  |  |  |
| Rural | 1 (Reference) | 1 (Reference) | 1 (Reference) | 1 (Reference) | 1 (Reference) | 1 (Reference) |
| Urban | **1.41 (1.29-1.54)** | **1.76 (1.60-1.93)** | **1.42 (1.13-1.77)** | **1.33 (1.05-1.67)** | 0.80 (0.64-1.01) | 0.97 (0.77-1.22) |
| **Neonatal factors** |  |  |  |  |  |  |
| *Birthweight, n(%)* |  |  |  |  |  |  |
| Low birthweight | 1 (Reference) | 1 (Reference) | 1 (Reference) | 1 (Reference) | 1 (Reference) | 1 (Reference) |
| Normal birthweight | 0.88 (0.71-1.08) | 0.87 (0.70-1.08) | 1.33 (0.65-2.70) | 1.41 (0.69-2.87) | 0.83 (0.48-1.44) | 0.75 (0.43-1.31) |
| High birthweight | **0.71 (0.55-0.91)** | **0.66 (0.50-0.85)** | **2.95 (1.39-6.26)** | **3.29 (1.55-7.01)** | 1.40 (0.76-2.59) | 1.28 (0.69-2.38) |
| *Breastfeeding duration, n(%)* |  |  |  |  |  |  |
| Non-breastfeeding | 1 (Reference) | 1 (Reference) | 1 (Reference) | 1 (Reference) | 1 (Reference) | 1 (Reference) |
| 0-6 months | **1.22 (1.07-1.39)** | **1.22 (1.06-1.39)** | 1.18 (0.83-1.67) | 1.21 (0.85-1.73) | 1.11 (0.79-1.54) | 1.12 (0.80-1.56) |
| 6-12 months | **1.37 (1.20-1.56)** | **1.39 (1.22-1.59)** | 0.87 (0.60-1.25) | 0.93 (0.64-1.34) | 0.88 (0.63-1.24) | 0.90 (0.64-1.27) |
| ＞12 months | **1.18 (1.01-1.38)** | **1.30 (1.11-1.53)** | **1.72 (1.18-2.50)** | **1.92 (1.31-2.80)** | 1.31 (0.90-1.89) | 1.38 (0.94-2.02) |
| **Parental or family factors** |  |  |  |  |  |  |
| *Paternal weight status* |  |  |  |  |  |  |
| Normal | 1 (Reference) | 1 (Reference) | 1 (Reference) | 1 (Reference) | 1 (Reference) | 1 (Reference) |
| Overweight | **1.21 (1.11-1.33)** | **1.21 (1.10-1.33)** | **1.96 (1.49-2.58)** | **1.94 (1.48-2.56)** | **2.09 (1.61-2.70)** | **2.15 (1.66-2.78)** |
| Obesity | **1.18 (1.03-1.36)** | **1.24 (1.07-1.44)** | **4.91 (3.64-6.62)** | **4.67 (3.46-6.31)** | **3.93 (2.91-5.32)** | **4.30 (3.17-5.83)** |
| *Maternal weight status* |  |  |  |  |  |  |
| Normal | 1 (Reference) | 1 (Reference) | 1 (Reference) | 1 (Reference) | 1 (Reference) | 1 (Reference) |
| Overweight | **1.12 (1.01-1.25)** | 1.08 (0.97-1.21) | **2.17 (1.67-2.82)** | **2.35 (1.80-3.06)** | **2.45 (1.90-3.15)** | **2.48 (1.92-3.19)** |
| Obesity | 1.01 (0.81-1.27) | 1.01 (0.80-1.27) | **3.53 (2.36-5.28)** | **3.92 (2.61-5.87)** | **4.19 (2.89-6.09)** | **4.26 (2.92-6.23)** |
| *Paternal educational attainment* |  |  |  |  |  |  |
| Primary school or below | 1 (Reference) | 1 (Reference) | 1 (Reference) | 1 (Reference) | 1 (Reference) | 1 (Reference) |
| Secondary or equivalent | **0.78 (0.67-0.92)** | **0.78 (0.66-0.92)** | 1.29 (0.76-2.19) | 1.11 (0.65-1.90) | 1.16 (0.72-1.87) | 1.15 (0.71-1.87) |
| Junior college or above | **0.60 (0.50-0.71)** | **0.59 (0.49-0.72)** | 1.37 (0.79-2.38) | 0.96 (0.54-1.70) | 0.89 (0.53-1.47) | 0.91 (0.54-1.55) |
| *Maternal educational attainment* |  |  |  |  |  |  |
| Primary school or below | 1 (Reference) | 1 (Reference) | 1 (Reference) | 1 (Reference) | 1 (Reference) | 1 (Reference) |
| Secondary or equivalent | **0.77 (0.67-0.89)** | **0.78 (0.67-0.91)** | 1.05 (0.68-1.64) | 0.88 (0.56-1.38) | 1.17 (0.77-1.79) | 1.16 (0.76-1.78) |
| Junior college or above | **0.65 (0.55-0.76)** | **0.67 (0.57-0.80)** | 1.32 (0.83-2.09) | 0.90 (0.55-1.47) | 0.89 (0.56-1.41) | 0.91 (0.56-1.49) |
| *Monthly household income* |  |  |  |  |  |  |
| < 5000 yuan | 1 (Reference) | 1 (Reference) | 1 (Reference) | 1 (Reference) | 1 (Reference) | 1 (Reference) |
| 5000-12000 yuan | 0.91 (0.82-1.01) | 0.91 (0.82-1.01) | 1.01 (0.78-1.32) | 0.95 (0.73-1.23) | 0.85 (0.66-1.11) | 0.87 (0.67-1.12) |
| ≥12000 yuan | **0.74 (0.62-0.89)** | **0.76 (0.63-0.91)** | **0.48 (0.27-0.85)** | **0.41 (0.23-0.73)** | **0.50 (0.29-0.85)** | **0.53 (0.31-0.91)** |
| Model: adjusted for age, sex, single-child status and residence area. | | | | | | |
| *MHNW was regarded as the reference group, and the metabolically obesity phenotype was defined by CMRFs criteria. | | | | | | |
| Bold values referred to P <0.05. | | | | | | |

| Supplementary Table 5. Odds ratios for different metabolic body size phenotype associated with demographic, neonatal, parental or family factors, based on MetS components criteria. | | | | | | |
| --- | --- | --- | --- | --- | --- | --- |
| Characteristics | MUNW* | | MHO* | | MUO* | |
|  | Unadjusted OR (95% CI) | Model OR (95% CI) | Unadjusted OR (95% CI) | Model OR (95% CI) | Unadjusted OR (95% CI) | Model OR (95% CI) |
|  |  |  |  |  |  |  |
| **Demographic factors** |  |  |  |  |  |  |
| *Age* |  |  |  |  |  |  |
| 7-12 years | 1 (Reference) | 1 (Reference) | 1 (Reference) | 1 (Reference) | 1 (Reference) | 1 (Reference) |
| 13-18 years | **1.70 (1.48-1.95)** | **1.91 (1.65-2.20)** | 0.92 (0.80-1.07) | 0.90 (0.78-1.04) | **1.42 (1.15-1.76)** | **1.37 (1.10-1.70)** |
| *Sex* |  |  |  |  |  |  |
| Boy | 1 (Reference) | 1 (Reference) | 1 (Reference) | 1 (Reference) | 1 (Reference) | 1 (Reference) |
| Girl | 1.01 (0.88-1.16) | 0.98 (0.85-1.13) | 1.10 (0.96-1.26) | **1.13 (1.00-1.30)** | **0.80 (0.65-0.99)** | **0.80 (0.65-0.99)** |
| *Single-child status* |  |  |  |  |  |  |
| No | 1 (Reference) | 1 (Reference) | 1 (Reference) | 1 (Reference) | 1 (Reference) | 1 (Reference) |
| Yes | **0.83 (0.72-0.95)** | **0.81 (0.70-0.94)** | **1.35 (1.16-1.58)** | **1.38 (1.18-1.61)** | **1.30 (1.02-1.64)** | 1.25 (0.99-1.59) |
| *Residence area* |  |  |  |  |  |  |
| Rural | 1 (Reference) | 1 (Reference) | 1 (Reference) | 1 (Reference) | 1 (Reference) | 1 (Reference) |
| Urban | **1.79 (1.54-2.08)** | **2.09 (1.79-2.44)** | **1.27 (1.10-1.46)** | **1.19 (1.03-1.38)** | **0.76 (0.62-0.94)** | 0.84 (0.67-1.04) |
| **Neonatal factors** |  |  |  |  |  |  |
| *Birthweight, n(%)* |  |  |  |  |  |  |
| Low birthweight | 1 (Reference) | 1 (Reference) | 1 (Reference) | 1 (Reference) | 1 (Reference) | 1 (Reference) |
| Normal birthweight | 0.77 (0.56-1.05) | 0.80 (0.58-1.10) | 1.37 (0.91-2.06) | 1.35 (0.90-2.04) | 1.19 (0.66-2.13) | 1.10 (0.61-1.97) |
| High birthweight | **0.66 (0.45-0.99)** | **0.64 (0.43-0.96)** | **2.58 (1.66-4.01)** | **2.71 (1.74-4.23)** | 1.43 (0.74-2.78) | 1.33 (0.69-2.59) |
| *Breastfeeding duration, n(%)* |  |  |  |  |  |  |
| Non-breastfeeding | 1 (Reference) | 1 (Reference) | 1 (Reference) | 1 (Reference) | 1 (Reference) | 1 (Reference) |
| 0-6 months | 1.13 (0.92-1.39) | 1.07 (0.86-1.32) | 1.07 (0.87-1.31) | 1.07 (0.87-1.32) | 0.93 (0.68-1.27) | 0.94 (0.69-1.29) |
| 6-12 months | **1.46 (1.21-1.77)** | **1.44 (1.19-1.75)** | 0.89 (0.73-1.10) | 0.91 (0.74-1.12) | 0.82 (0.60-1.12) | 0.85 (0.62-1.16) |
| ＞12 months | **1.50 (1.21-1.87)** | **1.53 (1.22-1.91)** | **1.34 (1.06-1.68)** | **1.37 (1.09-1.73)** | 1.23 (0.87-1.74) | 1.27 (0.89-1.80) |
| **Parental or family factors** |  |  |  |  |  |  |
| *Paternal weight status* |  |  |  |  |  |  |
| Normal | 1 (Reference) | 1 (Reference) | 1 (Reference) | 1 (Reference) | 1 (Reference) | 1 (Reference) |
| Overweight | 1.10 (0.95-1.27) | 1.08 (0.93-1.25) | **1.74 (1.49-2.03)** | **1.75 (1.50-2.04)** | **1.88 (1.48-2.39)** | **1.93 (1.52-2.45)** |
| Obesity | 0.97 (0.76-1.23) | 0.97 (0.76-1.24) | **3.18 (2.64-3.84)** | **3.22 (2.66-3.89)** | **3.11 (2.32-4.17)** | **3.32 (2.47-4.46)** |
| *Maternal weight status* |  |  |  |  |  |  |
| Normal | 1 (Reference) | 1 (Reference) | 1 (Reference) | 1 (Reference) | 1 (Reference) | 1 (Reference) |
| Overweight | 1.03 (0.86-1.23) | 1.01 (0.84-1.21) | **1.74 (1.48-2.05)** | **1.82 (1.55-2.14)** | **1.99 (1.57-2.54)** | **2.02 (1.59-2.58)** |
| Obesity | 0.90 (0.61-1.34) | 0.92 (0.62-1.37) | **2.89 (2.23-3.74)** | **3.06 (2.35-3.98)** | **2.84 (1.90-4.24)** | **2.94 (1.96-4.41)** |
| *Paternal educational attainment* |  |  |  |  |  |  |
| Primary school or below | 1 (Reference) | 1 (Reference) | 1 (Reference) | 1 (Reference) | 1 (Reference) | 1 (Reference) |
| Secondary or equivalent | 0.92 (0.71-1.20) | 0.89 (0.68-1.16) | 1.43 (0.98-2.00) | 1.29 (0.92-1.81) | 1.39 (0.87-2.24) | 1.37 (0.85-2.21) |
| Junior college or above | **0.66 (0.50-0.88)** | **0.60 (0.44-0.82)** | **1.58 (1.12-2.23)** | 1.24 (0.86-1.77) | 1.18 (0.71-1.94) | 1.19 (0.71-2.01) |
| *Maternal educational attainment* |  |  |  |  |  |  |
| Primary school or below | 1 (Reference) | 1 (Reference) | 1 (Reference) | 1 (Reference) | 1 (Reference) | 1 (Reference) |
| Secondary or equivalent | 0.93 (0.74-1.18) | 0.92 (0.72-1.17) | 1.12 (0.87-1.45) | 1.06 (0.81-1.37) | 1.40 (0.92-2.12) | 1.37 (0.89-2.09) |
| Junior college or above | **0.71 (0.54-0.92)** | **0.67 (0.50-0.89)** | 1.32 (1.00-1.73) | 1.17 (0.88-1.56) | 1.15 (0.73-1.80) | 1.14 (0.71-1.83) |
| *Monthly household income* |  |  |  |  |  |  |
| < 5000 yuan | 1 (Reference) | 1 (Reference) | 1 (Reference) | 1 (Reference) | 1 (Reference) | 1 (Reference) |
| 5000-12000 yuan | 0.86 (0.73-1.02) | 0.85 (0.72-1.00) | 1.05 (0.90-1.23) | 1.03 (0.88-1.21) | 0.97 (0.76-1.24) | 0.98 (0.77-1.26) |
| ≥12000 yuan | **0.69 (0.51-0.94)** | **0.67 (0.50-0.92)** | 0.95 (0.72-1.24) | 0.91 (0.70-1.20) | **0.52 (0.31-0.88)** | **0.55 (0.32-0.92)** |
| Model: adjusted for age, sex, single-child status and residence area. | | | | | | |
| *MHNW was regarded as the reference group, and the metabolically obesity phenotype was defined by MetS components criteria. | | | | | | |
| Bold values referred to P <0.05. | | | | | | |

| Supplementary Table 6. Odds ratios for MUO associated with lifestyle factors, with the reference of MHO. | | | | | | |
| --- | --- | --- | --- | --- | --- | --- |
| Characteristics | 2018 consensus-based criteria-MUO* | | CMRFs criteria-MUO* | | MetS components criteria-MUO* | |
|  | Unadjusted | Model | Unadjusted | Model | Unadjusted | Model |
|  | OR (95% CI) | OR (95% CI) | OR (95% CI) | OR (95% CI) | OR (95% CI) | OR (95% CI) |
| **Lifestyle factors** |  |  |  |  |  |  |
| *Fruit Consumption* |  |  |  |  |  |  |
| < 0.75 serving /day | 1 (Reference) | 1 (Reference) | 1 (Reference) | 1 (Reference) | 1 (Reference) | 1 (Reference) |
| 0.75-1.5 serving /day | 1.11 (0.74-1.65) | 1.16 (0.77-1.75) | 0.99 (0.67-1.46) | 1.05 (0.69-1.60) | 0.81 (0.60-1.11) | 0.85 (0.62-1.16) |
| ≥ 1.5 serving /day | 1.05 (0.71-1.56) | 1.16 (0.77-1.75) | 1.22 (0.83-1.80) | 1.46 (0.96-2.22) | 1.17 (0.87-1.57) | 1.25 (0.92-1.69) |
| *Vegetable Consumption* |  |  |  |  |  |  |
| < 1 serving /day | 1 (Reference) | 1 (Reference) | 1 (Reference) | 1 (Reference) | 1 (Reference) | 1 (Reference) |
| 1-3 serving /day | 1.27 (0.85-1.90) | 1.21 (0.80-1.84) | 1.47 (0.98-2.18) | 1.31 (0.85-2.01) | 1.23 (0.90-1.70) | 1.21 (0.87-1.67) |
| ≥ 3 serving /day | 1.27 (0.78-2.08) | 1.22 (0.74-2.01) | **1.84 (1.13-2.99)** | **1.72 (1.03-2.89)** | 1.39 (0.95-2.02) | 1.34 (0.91-1.96) |
| *SSB consumption* |  |  |  |  |  |  |
| 0 serving /day | 1 (Reference) | 1 (Reference) | 1 (Reference) | 1 (Reference) | 1 (Reference) | 1 (Reference) |
| < 1 serving /day | 1.19 (0.82-1.72) | 1.09 (0.74-1.59) | 1.44 (0.99-2.07) | 1.35 (0.91-1.99) | 0.83 (0.62-1.11) | 0.80 (0.59-1.07) |
| ≥ 1 servings /day | **1.65 (1.08-2.53)** | 1.28 (0.82-1.99) | **2.15 (1.42-3.25)** | 1.52 (0.97-2.39) | 1.21 (0.89-1.65) | 0.93 (0.67-1.28) |
| *Sedentary time* |  |  |  |  |  |  |
| ≤ 3 hours /day | 1 (Reference) | 1 (Reference) | 1 (Reference) | 1 (Reference) | 1 (Reference) | 1 (Reference) |
| 3-7 hours /day | 0.87 (0.52-1.47) | 0.85 (0.50-1.45) | 0.85 (0.51-1.43) | 0.79 (0.46-1.38) | 0.90 (0.59-1.37) | 0.92 (0.60-1.41) |
| ≥ 7 hours /day | 1.04 (0.74-1.47) | 1.04 (0.73-1.50) | 1.01 (0.72-1.41) | 0.91 (0.63-1.32) | 0.96 (0.74-1.24) | 0.86 (0.65-1.13) |
| *Screen time* |  |  |  |  |  |  |
| < 1 hours /day | 1 (Reference) | 1 (Reference) | 1 (Reference) | 1 (Reference) | 1 (Reference) | 1 (Reference) |
| 1-3 hours /day | 0.68 (0.47-1.01) | 0.70 (0.48-1.01) | 0.74 (0.52-1.05) | 0.81 (0.55-1.18) | 0.80 (0.61-1.06) | 0.84 (0.63-1.11) |
| ≥ 3 hours /day | 0.66 (0.41-1.04) | 0.67 (0.44-1.02) | 0.68 (0.43-1.07) | 0.72 (0.45-1.15) | 0.96 (0.67-1.37) | 0.90 (0.63-1.29) |
| *Sleep duration* |  |  |  |  |  |  |
| < 7 hours | 1 (Reference) | 1 (Reference) | 1 (Reference) | 1 (Reference) | 1 (Reference) | 1 (Reference) |
| 7-9 hours | **0.58 (0.37-0.91)** | 0.74 (0.46-1.20) | **0.52 (0.34-0.81)** | 0.84 (0.52-1.36) | 0.95 (0.68-1.32) | 1.20 (0.84-1.70) |
| ≥ 9 hours | **0.58 (0.34-0.99)** | 0.90 (0.49-1.64) | **0.39 (0.23-0.67)** | 0.89 (0.49-1.63) | 0.81 (0.53-1.22) | 1.27 (0.80-2.01) |
| *Physical activity* |  |  |  |  |  |  |
| 0 hours /day | 1 (Reference) | 1 (Reference) | 1 (Reference) | 1 (Reference) | 1 (Reference) | 1 (Reference) |
| < 0.5 hours /day | 0.87 (0.60-1.24) | 1.04 (0.71-1.53) | **0.68 (0.47-0.97)** | 0.94 (0.64-1.39) | **0.75 (0.57-0.99)** | 0.86 (0.64-1.15) |
| ≥ 0.5 hours /day | 1.17 (0.75-1.84) | 1.20 (0.75-1.90) | 1.01 (0.65-1.55) | 1.12 (0.70-1.79) | 0.87 (0.63-1.21) | 0.90 (0.64-1.25) |
| Model: adjusted for age, sex, single-child status and residence area. | | | | | | |
| *MHO was regarded as the reference group. | | | | | | |
| Bold values referred to P <0.05. | | | | | | |

| Supplementary Table 7. Odds ratios for different metabolic body size phenotype associated with lifestyle factors, based on CMRFs criteria. | | | | | | |
| --- | --- | --- | --- | --- | --- | --- |
| Characteristics | MUNW* | | MHO* | | MUO* | |
|  | Unadjusted OR (95% CI) | Model OR (95% CI) | Unadjusted OR (95% CI) | Model OR (95% CI) | Unadjusted OR (95% CI) | Model OR (95% CI) |
|  |  |  |  |  |  |  |
| **Lifestyle factors** |  |  |  |  |  |  |
| *Fruit Consumption* |  |  |  |  |  |  |
| < 0.75 serving /day | 1 (Reference) | 1 (Reference) | 1 (Reference) | 1 (Reference) | 1 (Reference) | 1 (Reference) |
| 0.75-1.5 serving /day | **0.89 (0.81-0.99)** | 0.94 (0.85-1.04) | 1.04 (0.79-1.37) | 1.03 (0.78-1.36) | 1.03 (0.78-1.36) | 1.07 (0.81-1.42) |
| ≥ 1.5 serving /day | 0.95 (0.86-1.06) | 0.99 (0.89-1.11) | 1.16 (0.87-1.54) | 1.12 (0.84-1.49) | **1.41 (1.08-1.86)** | **1.50 (1.14-1.97)** |
| *Vegetable Consumption* |  |  |  |  |  |  |
| < 1 serving /day | 1 (Reference) | 1 (Reference) | 1 (Reference) | 1 (Reference) | 1 (Reference) | 1 (Reference) |
| 1-3 serving /day | 0.99 (0.89-1.10) | 0.96 (0.87-1.07) | 0.89 (0.67-1.16) | 0.87 (0.66-1.15) | 1.30 (0.96-1.75) | 1.28 (0.95-1.73) |
| ≥ 3 serving /day | 1.00 (0.88-1.14) | 0.93 (0.82-1.07) | 0.84 (0.59-1.20) | 0.86 (0.60-1.22) | **1.55 (1.09-2.19)** | **1.50 (1.06-2.12)** |
| *SSB consumption* |  |  |  |  |  |  |
| 0 serving /day | 1 (Reference) | 1 (Reference) | 1 (Reference) | 1 (Reference) | 1 (Reference) | 1 (Reference) |
| < 1 serving /day | 0.88 (0.80-1.00) | **0.87 (0.78-0.96)** | 0.84 (0.65-1.09) | 0.85 (0.66-1.10) | 1.20 (0.92-1.58) | 1.18 (0.90-1.54) |
| ≥ 1 servings /day | **1.16 (1.04-1.30)** | 0.94 (0.84-1.06) | 0.84 (0.62-1.15) | 1.00 (0.73-1.38) | **1.81 (1.37-2.41)** | **1.46 (1.08-1.95)** |
| *Sedentary time* |  |  |  |  |  |  |
| ≤ 3 hours /day | 1 (Reference) | 1 (Reference) | 1 (Reference) | 1 (Reference) | 1 (Reference) | 1 (Reference) |
| 3-7 hours /day | 1.04 (0.93-1.16) | 1.02 (0.91-1.15) | 1.32 (0.91-1.91) | 1.29 (0.89-1.86) | 1.12 (0.77-1.63) | 1.12 (0.76-1.63) |
| ≥ 7 hours /day | 1.10 (0.99-1.23) | 0.89 (0.80-1.00) | 1.21 (0.95-1.55) | 1.27 (0.99-1.64) | 1.22 (0.96-1.55) | 1.03 (0.81-1.32) |
| *Screen time* |  |  |  |  |  |  |
| < 1 hours /day | 1 (Reference) | 1 (Reference) | 1 (Reference) | 1 (Reference) | 1 (Reference) | 1 (Reference) |
| 1-3 hours /day | **0.79 (0.72-0.87)** | **0.86 (0.78-0.95)** | 1.24 (0.96-1.61) | 1.26 (0.97-1.64) | 0.92 (0.71-1.18) | 0.98 (0.76-1.26) |
| ≥ 3 hours /day | 0.89 (0.78-1.01) | 0.87 (0.76-1.00) | **1.47 (1.06-2.03)** | **1.68 (1.21-2.34)** | 0.99 (0.71-1.39) | 0.93 (0.66-1.30) |
| *Sleep duration* |  |  |  |  |  |  |
| < 7 hours | 1 (Reference) | 1 (Reference) | 1 (Reference) | 1 (Reference) | 1 (Reference) | 1 (Reference) |
| 7-9 hours | **0.64 (0.57-0.71)** | **0.84 (0.75-0.94)** | 1.25 (0.88-1.78) | 1.01 (0.70-1.47) | **0.66 (0.50-0.87)** | 0.84 (0.63-1.13) |
| ≥ 9 hours | **0.41 (0.36-0.48)** | **0.71 (0.60-0.83)** | 1.23 (0.82-1.84) | 0.87 (0.57-1.35) | **0.48 (0.33-0.70)** | 0.79 (0.52-1.19) |
| *Physical activity* |  |  |  |  |  |  |
| 0 hours /day | 1 (Reference) | 1 (Reference) | 1 (Reference) | 1 (Reference) | 1 (Reference) | 1 (Reference) |
| < 0.5 hours /day | **0.81 (0.74-0.90)** | **0.91 (0.82-0.99)** | **0.75 (0.58-0.96)** | **0.74 (0.57-0.95)** | 0.94 (0.73-1.21) | 1.09 (0.85-1.42) |
| ≥ 0.5 hours /day | 0.90 (0.80-1.01) | 0.99 (0.87-1.11) | **0.68 (0.51-0.91)** | **0.69 (0.52-0.92)** | 1.14 (0.85-1.53) | 1.28 (0.95-1.73) |
| Model: adjusted for age, sex, single-child status and residence area. | | | | | | |
| *MHNW was regarded as the reference group, and the metabolically obesity phenotype was defined by CMRFs criteria. | | | | | | |
| Bold values referred to P <0.05. | | | | | | |

| Supplementary Table 8. Odds ratios for different metabolic body size phenotype associated with lifestyle factors, based on MetS components criteria. | | | | | | |
| --- | --- | --- | --- | --- | --- | --- |
| Characteristics | MUNW* | | MHO* | | MUO* | |
|  | Unadjusted OR (95% CI) | Model OR (95% CI) | Unadjusted OR (95% CI) | Model OR (95% CI) | Unadjusted OR (95% CI) | Model OR (95% CI) |
|  |  |  |  |  |  |  |
| **Lifestyle factors** |  |  |  |  |  |  |
| *Fruit Consumption* |  |  |  |  |  |  |
| < 0.75 serving /day | 1 (Reference) | 1 (Reference) | 1 (Reference) | 1 (Reference) | 1 (Reference) | 1 (Reference) |
| 0.75-1.5 serving /day | **0.84 (0.71-0.99)** | 0.88 (0.75-1.05) | 1.11 (0.94-1.31) | 1.09 (0.93-1.29) | 0.91 (0.69-1.18) | 0.92 (0.71-1.20) |
| ≥ 1.5 serving /day | 0.97 (0.82-1.15) | 1.00 (0.84-1.18) | 1.17 (0.99-1.39) | 1.15 (0.97-1.37) | **1.36 (1.06-1.76)** | **1.42 (1.10-1.83)** |
| *Vegetable Consumption* |  |  |  |  |  |  |
| < 1 serving /day | 1 (Reference) | 1 (Reference) | 1 (Reference) | 1 (Reference) | 1 (Reference) | 1 (Reference) |
| 1-3 serving /day | 1.04 (0.87-1.23) | 1.02 (0.86-1.21) | 1.02 (0.86-1.20) | 1.01 (0.85-1.19) | 1.25 (0.95-1.66) | 1.24 (0.94-1.64) |
| ≥ 3 serving /day | 1.14 (0.93-1.41) | 1.07 (0.87-1.32) | 1.11 (0.90-1.36) | 1.11 (0.90-1.37) | **1.54 (1.11-2.13)** | **1.51 (1.09-2.09)** |
| *SSB consumption* |  |  |  |  |  |  |
| 0 serving /day | 1 (Reference) | 1 (Reference) | 1 (Reference) | 1 (Reference) | 1 (Reference) | 1 (Reference) |
| < 1 serving /day | 0.93 (0.79-1.09) | 0.93 (0.79-1.09) | 1.09 (0.94-1.28) | 1.10 (0.94-1.29) | 0.91 (0.71-1.17) | 0.90 (0.70-1.16) |
| ≥ 1 servings /day | **1.24 (1.04-1.48)** | 1.11 (0.92-1.33) | **1.25 (1.05-1.50)** | **1.33 (1.11-1.60)** | **1.52 (1.17-1.97)** | **1.29 (1.00-1.69)** |
| *Sedentary time* |  |  |  |  |  |  |
| ≤ 3 hours /day | 1 (Reference) | 1 (Reference) | 1 (Reference) | 1 (Reference) | 1 (Reference) | 1 (Reference) |
| 3-7 hours /day | 1.00 (0.86-1.15) | 0.97 (0.84-1.12) | 1.11 (0.89-1.40) | 1.09 (0.87-1.37) | 1.00 (0.70-1.44) | 0.98 (0.68-1.41) |
| ≥ 7 hours /day | 0.92 (0.79-1.06) | 0.83 (0.74-1.01) | 1.13 (0.97-1.30) | 1.13 (0.98-1.31) | 1.08 (0.86-1.35) | 0.97 (0.77-1.22) |
| *Screen time* |  |  |  |  |  |  |
| < 1 hours /day | 1 (Reference) | 1 (Reference) | 1 (Reference) | 1 (Reference) | 1 (Reference) | 1 (Reference) |
| 1-3 hours /day | **0.77 (0.68-0.87)** | **0.84 (0.74-0.95)** | **1.29 (1.11-1.50)** | **1.30 (1.11-1.51)** | 1.03 (0.81-1.31) | 1.06 (0.83-1.35) |
| ≥ 3 hours /day | 0.89 (0.75-1.05) | 0.86 (0.72-1.02) | **1.27 (1.04-1.55)** | **1.33 (1.08-1.63)** | 1.22 (0.90-1.65) | 1.13 (0.83-1.54) |
| *Sleep duration* |  |  |  |  |  |  |
| < 7 hours | 1 (Reference) | 1 (Reference) | 1 (Reference) | 1 (Reference) | 1 (Reference) | 1 (Reference) |
| 7-9 hours | **0.69 (0.58-0.83)** | 0.84 (0.70-1.01) | 1.04 (0.86-1.26) | 0.97 (0.80-1.19) | 0.98 (0.74-1.31) | 1.15 (0.86-1.55) |
| ≥ 9 hours | **0.49 (0.38-0.61)** | **0.69 (0.53-0.90)** | 0.91 (0.73-1.15) | 0.81 (0.63-1.04) | 0.74 (0.51-1.06) | 1.02 (0.68-1.52) |
| *Physical activity* |  |  |  |  |  |  |
| 0 hours /day | 1 (Reference) | 1 (Reference) | 1 (Reference) | 1 (Reference) | 1 (Reference) | 1 (Reference) |
| < 0.5 hours /day | 0.87 (0.75-1.02) | 0.95 (0.81-1.11) | 0.90 (0.76-1.06) | 0.88 (0.74-1.04) | **0.74 (0.58-0.94)** | **0.79 (0.62-9.99)** |
| ≥ 0.5 hours /day | 0.95 (0.79-1.15) | 1.05 (0.86-1.27) | 0.99 (0.82-1.18) | 1.00 (0.83-1.20) | 1.05 (0.80-1.39) | 1.05 (0.79-1.38) |
| Model: adjusted for age, sex, single-child status and residence area. | | | | | | |
| *MHNW was regarded as the reference group, and the metabolically obesity phenotype was defined by MetS components criteria. | | | | | | |
| Bold values referred to P <0.05. | | | | | | |


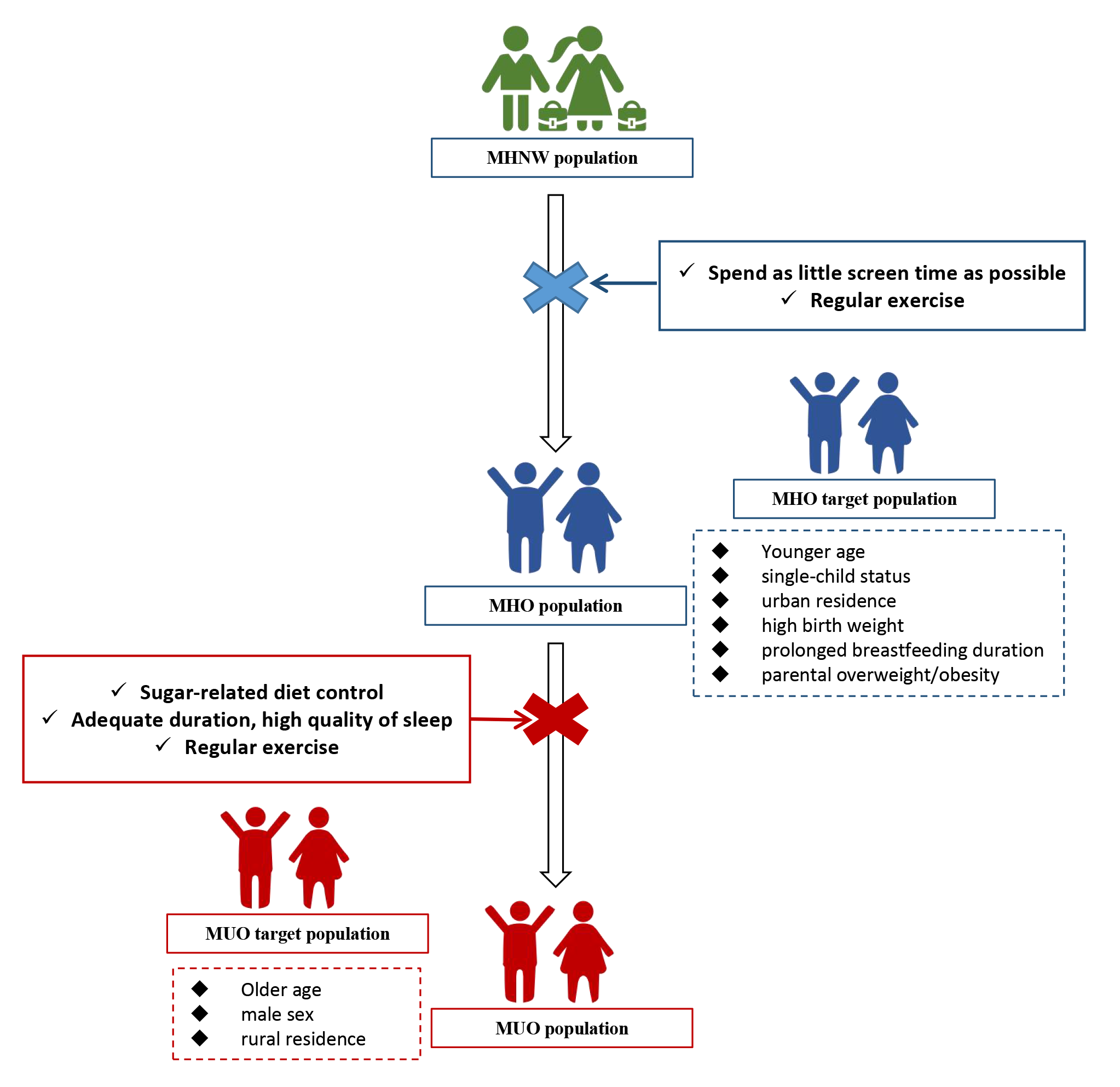


Supplementary Figure 1. Diagram of the different stages of prevention intervention strategy.
